# Insulin receptor activation by proinsulin preserves synapses and vision in retinitis pigmentosa

**DOI:** 10.1038/s41419-022-04839-0

**Published:** 2022-04-20

**Authors:** Alonso Sánchez-Cruz, Alberto Hernández-Pinto, Concepción Lillo, Carolina Isiegas, Miguel Marchena, Ignacio Lizasoain, Fátima Bosch, Pedro de la Villa, Catalina Hernández-Sánchez, Enrique J. de la Rosa

**Affiliations:** 1 Department of Molecular Biomedicine, Centro de Investigaciones Biológicas Margarita Salas (CSIC), C/ Ramiro de Maeztu 9, 28040 Madrid, Spain; 2 Neurovascular Research Unit, Department of Pharmacology and Toxicology and Instituto Universitario de Investigación en Neuroquímica, Facultad de Medicina, Universidad Complutense de Madrid, Ciudad Universitaria, 28040 Madrid, Spain; 3 Instituto de Investigación Hospital 12 de Octubre (IMAS12), 28041 Madrid, Spain; 4 Cell Biology and Pathology Department, University of Salamanca, Institute for Biomedical Research of Salamanca (IBSAL), Institute of Neurosciences of Castilla y León (INCYL), C/ Pintor Fernando Gallego,1, 37007 Salamanca, Spain; 5 Center of Animal Biotechnology and Gene Therapy and Department of Biochemistry and Molecular Biology, School of Veterinary Medicine, Universitat Autònoma de Barcelona, 08193 Bellaterra, Spain; 6 Centro de Investigación Biomédica en Red de Diabetes y Enfermedades Metabólicas Asociadas (CIBERDEM), ISCIII, 28034 Madrid, Spain; 7 Department of System Biology, Facultad de Medicina, Universidad de Alcalá, 28805 Alcalá de Henares, Spain; 8 Instituto Ramón y Cajal de Investigación Sanitaria, ISCIII, 28034 Madrid, Spain

**Keywords:** Neurodegeneration, Diseases

## Abstract

Synaptic loss, neuronal death, and circuit remodeling are common features of central nervous system neurodegenerative disorders. Retinitis pigmentosa (RP), the leading cause of inherited blindness, is a group of retinal dystrophies characterized by photoreceptor dysfunction and death. The insulin receptor, a key controller of metabolism, also regulates neuronal survival and synaptic formation, maintenance, and activity. Indeed, deficient insulin receptor signaling has been implicated in several brain neurodegenerative pathologies. We present evidence linking impaired insulin receptor signaling with RP. We describe a selective decrease in the levels of the insulin receptor and its downstream effector phospho-S6 in retinal horizontal cell terminals in the *rd10* mouse model of RP, as well as aberrant synapses between rod photoreceptors and the postsynaptic terminals of horizontal and bipolar cells. A gene therapy strategy to induce sustained proinsulin, the insulin precursor, production restored retinal insulin receptor signaling, by increasing S6 phosphorylation, without peripheral metabolic consequences. Moreover, proinsulin preserved photoreceptor synaptic connectivity and prolonged visual function in electroretinogram and optomotor tests. These findings point to a disease-modifying role of insulin receptor and support the therapeutic potential of proinsulin in retinitis pigmentosa.

## Introduction

Neurodegenerative disorders are complex pathological conditions that involve, among other processes, synaptic loss and neuronal cell death leading to deterioration of neuronal structure and function. According to the 2016 Global Burden of Disease report [1], neurodegenerative diseases are the second leading cause of death and a major cause of disability. The development of strategies to cure or at least delay the progression of neurodegenerative diseases has been hindered by their diverse etiology and complex nature, and by the limited regenerative capacity of neurons. There is thus an urgent need for effective medical interventions. As part of the central nervous system (CNS), the retina shares multiple pathophysiological features with the brain [2]. Despite their diverse etiology, retinal neurodegenerative diseases, like those affecting the brain, are characterized by synaptic failure and neuronal cell death [3]. Retinitis pigmentosa (RP) comprises a group of hereditary retinal neurodegenerative conditions with a complex genetic etiology. To date more than 60 genes and 3,000 mutations have been implicated in RP, and over 300 genes associated with inherited retinal dystrophies (https://sph.uth.edu/retnet/disease.htm) have been identified. RP is characterized by primary dysfunction and death of photoreceptor cells followed by reactive gliosis and remodeling of the retinal structure, resulting in vision loss and eventual blindness [3, 4]. RP is categorized as a rare disease (prevalence 1/3,500-4,000), but accounts for most cases of hereditary blindness. Gene therapy would be the ideal definitive treatment, and one such therapy has been recently approved for a related retinal dystrophy [5]. However, the complexity and diversity of the mutations underlying RP necessitate the development of alternative therapeutic approaches, particularly those independent of the causative mutation, as well as palliative treatments. The insulin receptor (INSR), traditionally considered a key peripheral metabolic regulator, is increasingly viewed as an important modulator of neuronal cell survival, and synaptic formation, maintenance, and activity [6–8]. Since initial reports described widespread INSR expression in the CNS, including the retina [9–13], INSR signaling has been implicated in a growing number of CNS functions. In addition to regulating feeding behavior and peripheral metabolism, central INSR signaling is involved in memory formation and cognitive functions [6, 7, 14]. The mechanisms underpinning the non-metabolic actions of INSR are being gradually unraveled. At the neuronal level, INSR is involved in the control of synaptic function, through regulation of neurotransmitter receptor trafficking, and in synapse maintenance and dendritic arbor formation [8, 15]. Downregulation of INSR signaling has been implicated in several neurodegenerative diseases [6], particularly Alzheimer’s disease [16–19], and INSR stimulation proposed as a potential treatment for neurodegenerative disorders [20].

We previously showed that INSR stimulation in the embryonic retina promotes neuronal differentiation and downregulates developmental cell death [21, 22], and that the insulin precursor proinsulin can slow RP progression [23–25]. In the present study, we investigated the role of INSR in retinal neurodegeneration and sought to characterize the neuroprotective role of proinsulin as a putative INSR ligand. We present the first evidence linking INSR downregulation with retinal neurodegeneration, and provide insight into the multifaceted neuroprotective role of INSR. We employed a gene therapy strategy to produce sustained increases in systemic proinsulin levels without peripheral metabolic consequences. Proinsulin treatment restored INSR signaling as measured by S6 phosphorylation, preserved photoreceptor synaptic connectivity, and more importantly, extended visual function in a murine model of RP.

## Materials and methods

### Animals

The *rd10* mouse is an autosomal recessive homozygous mutant carrying a mutation, identical to one of those found in human patients, in the phosphodiesterase 6b (*Pde6b*^*rd10/rd10*^) on a C57BL/6J background [26]. Both *rd10* and wild type (WT) control mice of the same background were obtained from The Jackson Laboratory (Bar Harbor, ME, USA). All animals were housed and handled in accordance with the 3Rs principle, the ARVO statement for the Use of Animals in Ophthalmic and Vision Research, European Union guidelines, and those of the local ethics committees of the CSIC and the Comunidad de Madrid (Spain). Mice were bred in the CIB core facilities on a 12/12-h light/dark cycle. Light intensity was maintained at 3–5 lx. Males and females were included in the study. The number of animals was selected to ensure statistical significance applying the Ethical Guidelines for Statistical Practice of the American Statistical Association. The animals were assigned randomly to each experimental group without any prior consideration.

### Generation and administration of adeno-associated viral vectors

Recombinant AAV serotype 2/1 viral vectors bearing cDNA from the human proinsulin (hPi) gene under control of the cytomegalovirus promoter (AAV-hPi) or without human proinsulin cDNA (AAV-null) were generated in the Center for Animal Biotechnology and Gene Therapy at the Universitat Autònoma de Barcelona, as previously described [27]. The AAV system requires ~7 days to achieve sustained levels of human proinsulin. Therefore, to ensure that human proinsulin was expressed from early stages of the degenerative process, the *rd10* mice had to be injected at P10-P12. We chose intramuscular vs intraocular injection to prevent further retinal damage caused by the forced opening of the eyes which are still close at the injection time. *rd10* mice received a single intramuscular injection of 7.2 × 10^11^ vector genomes/kg body weight of AAV-hPi or AAV-null at P10–12. The total vector dose was distributed equally between the gastrocnemius muscles of both hind limbs.

### Measurement of proinsulin, insulin, and glycaemia

Serum, eye, and retinal levels of human proinsulin and serum levels of human insulin were measured using human Pi and human insulin ELISA kits (EZHPI-15K and EZHI-14K, respectively; Millipore, Darmstadt, Germany) according to the manufacturer’s instructions and as described previously [25]. Glycaemia was directly measured in blood samples using the Glucocart™ Gmeter kit (A. Menarini Diagnostics Ltd., Berkshire, UK).

### RNA isolation and RT-PCR

Total RNA from tissues was isolated using Trizol reagent (Invitrogen). The reverse transcriptase reaction (RT) was typically performed with 2 µg RNA, the Superscript III Kit, and random primers (all from ThermoFisher Scientific, Waltham, MA), followed by amplification with the 2X PANGEA-Long PCR Master Mix (Canvax Biotech, Córdoba, Spain). For conventional PCR mouse *Insr* was amplified using the following primers: sense, 5′-GGCCAGTGAGTGCTGCTCATGC-3′ (inside exon 10); antisense, 5′-TGTGGTGGCTGTCACATTCC-3’ (inside exon 12). Mouse β-actin was amplified using the following primers: sense, 5′-AAGGCCAACCGTGAAAAGAT-3’; antisense, 5′-GTGGTACGACCAGAGGCATAC-3’. For quantitative (q)PCR before performing the RT reaction, total RNA was treated with DNAse I (ThermoFisher Scientific) to eliminate potential contaminating DNA. RT was performed with 1 µg RNA and also with the Superscript III Kit and random primers (ThermoFisher). qPCR was performed with the ABI Prism 7900HT Sequence Detection System using Taq-Man Universal PCR Master Mix, no-AmpEThrase UNG, and Taqman assays (all from ThermoFisher): for *Rps6* (Mm02342456_g1); for *Insr* (Mm01211875_m1) and for *Tbp* (Mm01277042_m1).

### Immunofluorescence and image analysis

Animals were euthanized and their eyes enucleated and fixed for 50 min in freshly prepared 4% (w/v) paraformaldehyde in Sörensen’s phosphate buffer (SPB) (0.1 M, pH 7.4), and then cryoprotected by incubation in increasing concentrations of sucrose [final concentration, 50% (w/v) in SPB]. The eyes were then embedded in Tissue-Tek OCT (Sakura Finetec, Torrance, CA, USA) and snap frozen in dry-ice cold isopentane. Equatorial sections (12 µm) were cut on a cryostat and mounted on Superfrost Plus slides (ThermoFisher Scientific), dried at room temperature, and stored at −20 °C until the day of the assay.

Before performing further analyses, slides were dried at room temperature. After rinsing in PBS and permeation with 0.2% (w/v) Triton X-100 in PBS, sections were incubated with blocking buffer [5% (v/v) normal goat serum, 1% (w/v) Tween-20, 1 M glycine in PBS] for 1 h and then incubated overnight at 4 °C with primary antibodies (Table S1) diluted in blocking buffer. Sections incubated in the absence of primary antibody were used as specificity controls. After rinsing in PBS and incubation with the appropriate secondary antibodies (Table S1), sections were stained with DAPI (4´,6-diamidino-2-phenylindole; Sigma-Aldrich Corp., St. Louis, MO, USA) and cover slipped with Fluoromount-G (ThermoFisher Scientific). For GluA2 and mGluR6 immunostaining, an antigen retrieval step was performed prior to incubation in blocking buffer. To this end, sections were incubated in citrate buffer [10 mM sodium citrate, 0.05% (w/v) Tween-20, pH 6.0] in boiling water for 10 min. After cooling to room temperature, sections were rinsed with PBS and then incubated in freshly prepared 0.2% (w/v) sodium borohydride in PBS before continuing with the blocking reaction described above.

Sections were analyzed using a laser confocal microscope (TCS SP5 and TCS SP8; Leica Microsystems, Wetzlar, Germany). In all cases, retinal sections to be compared were stained and imaged under identical conditions. To measure the area occupied by INSR and NF-M immunostaining, images were converted to black and white and analyzed with Fiji software. To quantify the number of horizontal cell tips with associated pS6^Ser240/244^ punctate staining, over 200 horizontal cell tips per animal in 4 retinal areas were analyzed. Similarly, for synapse quantification, over 200 rod ribbons per animal in 4 retinal areas were assessed for associated GluA2 (horizontal cell postsynaptic terminal) or mGluR6 (bipolar cell postsynaptic terminal) punctate immunostaining. To measure the levels of S6 ribosomal protein in the OPL, the mean intensity of S6 fluorescence was quantified in 4 predefined 10 µm^2^ areas for each image. Four images per mouse were analyzed. To evaluate photoreceptor preservation in the whole retina, three sections per retina were analyzed: for each section, six areas in a nasotemporal sequence were photographed [28] (Fig. S1). ONL and INL thickness were measured in three random positions for each image. To evaluate the preservation of the outer segments sections three sections per retina were analyzed: for each section, two areas were photographed (T1 and T6) and cone and rod OS were measured in three random positions for each image. The value for each retina was considered the mean of T1 and T6 areas. When possible image analysis was performed by a researcher blinded to the experimental conditions.

### Transmission electron microscopy

Animals were euthanized and their eyes enucleated and fixed overnight in freshly prepared 2% (w/v) paraformaldehyde and 2% (w/v) glutaraldehyde in sodium cacodylate buffer (0.1 M, pH 7.4). After dissection of the cornea and lens, optic cups were post-fixed for 1 h with 1% (w/v) OsO_4_ and 1% (w/v) K_3_Fe(CN)_6_ in ultrapure water, dehydrated through a graded series of ethanol solutions and embedded in Epoxy EMbed-812 resin (EMS, Electron Microscopy Sciences). Semi-thin (0.5 μm) and ultra-thin sections were obtained using an Ultracut E ultramicrotome (Leica). Semi-thin sections were stained with Toluidine Blue and mounted with Entellan and images were obtained using a light microscope (Axio Observer Z1, Zeiss) with 20× and 100× oil immersion objectives. Ultra-thin sections were contrasted with uranyl acetate and lead citrate, and analyzed at the NUCLEUS electron microscopy facility at the University of Salamanca using a Tecnai Spirit Twin 120 kv electron microscope with a CCD Gatan Orius SC200D camera with DigitalMicrograph™ software. The proportions of the different types of synapses were quantified in ultra-thin sections from 3 mice of each genotype by analyzing between 58 and 86 synapses per mouse.

### ERG recordings and optomotor tests

Mice were handled and ERGs performed as previously described [29]. Measurements and data analysis were performed by two independent observers, both of them blind to experimental condition. Mice were maintained in darkness overnight. The next day the animals were anesthetized in scotopic conditions with ketamine (50 mg/kg; Ketolar, Pfizer, New York, NY) and medetomidine (0.3 mg/kg; Domtor, Orion Corporation, Espoo, Finland), and their pupils dilated with a drop of tropicamide (Alcon, Fort Worth, TX, USA). Then, the ground electrode was located parallel to the tail of the animal and the reference electrode was placed in the mouth. Methocel (Colorcon, Harleysville, PA, USA) was applied to the cornea to avoid drying and the corneal electrode was placed in contact with the Methocel. After ERG recording, sedation was interrupted with atipamezol (1 mg/kg; Antisedan, Orion Corporation). ERG responses were recorded using a device designed by Dr. P. de la Villa (Universidad de Alcala, Madrid, Spain). ERG recordings were first obtained in scotopic conditions and the ERG signals were amplified and band filtered between 0.3 and 1000 Hz (CP511 Preamplifier, Grass Instruments, Quincy, MA, USA) and digitized to 10 kHz using a PowerLab acquisition data card (AD Instruments Ltd., Oxfordshire, UK). Graphical representations of the signals recorded and luminous stimuli control were performed with Scope v6.4 PowerLab software (AD Instruments, Oxford, UK). ERG wave amplitudes were measured off-line and the results averaged. Wave amplitude analysis was performed using the MATLAB application.

An optomotor device was built based on the design proposed by Prusky et al. [30]. Measurements and data analysis were performed by two independent observers, both of them blind to experimental condition. Mice were placed in the center of a square array of computer monitors that displayed stimulus gratings, and then monitored using an overhead infrared television camera placed above the testing chamber. The test started with the most easily visible stimulus, with a spatial frequency of 0.088 cycles/degree, a temporal frequency of 0.88 Hz, and a normalized contrast of 1. Contrast sensitivity was calculated as the inverse of contrast threshold, and was measured at distinct spatial frequencies ranging from 0.022 to 0.355 cycles/degree (see below Fig. 8B). The Vision Egg tool was used for light stimulation. Stimuli consisted of vertical black/white bars (gratings) moving through the screens.

### Cell death analysis

Cell death in retinal sections was assessed by TUNEL assay (DeadEnd Flurometric TUNEL System, Promega, Madison, WI, USA, G3250) following the manufacturer’s instructions.

### Statistical analysis

Statistical analysis was performed with GraphPad Prism software 8.0 (GraphPad Software Inc., La Jolla, CA, USA). No samples were discarded from the analysis.

To compare two groups, normality was assessed for each group using the Shapiro-Wilk normality test. Normally distributed data were analyzed using an unpaired two-tailed T-test, and non-normally distributed data using a two-tailed Mann Whitney U-test. Homocedasticity for all data sets was assessed employing F test. When variance between data sets was significantly different Welch’s correction was applied to the T-test. Unpaired T-tests were used to compare insulin receptor and pS6^Ser240/244^ levels between WT and *rd10* animals, and to assess rod-horizontal and rod-bipolar connectivity in AAV-null and AAV-hPi mice.

Analysis of variables over time was achieved using a 1-way ANOVA. Dunnett’s multiple comparison test was used to compare values at different time points with those at a specific time point. Accordingly, serum, eye and retinal human proinsulin levels at different time points were compared with initials values using Dunnett’s multiple comparison test.

Comparisons of two variables were performed using a 2-way ANOVA, followed by Sidak’s multiple comparison test when a significant interaction between both variables was detected. Thus, Sidak’s multiple comparison test was used to compare the different synaptic morphologies between WT and *rd10* animals, and between AAV-null and AAV-hPi mice. Differences in histological parameters (ONL/INL ratio), ERG wave amplitude, and optomotor test results between AAV-null mice and AAV-hPi mice were assessed using a 2-way ANOVA.

In all cases, statistical significance was set at *p* ≤ 0.05.

## Results

### Insulin receptor expression and signaling in WT and rd10 mouse retinas

**Fig. 1:**
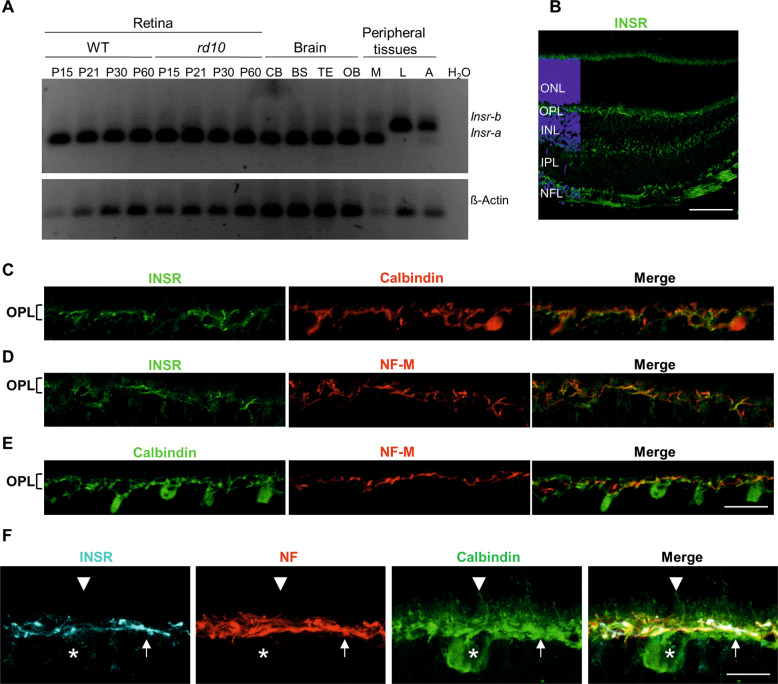
Insulin receptor expression in the mouse retina. **A** RT-PCR of WT and *rd10* mouse retinas at the indicated ages, and WT adult brain regions and peripheral tissues. Forward and reverse PCR primers corresponded to exons 10 and 12, respectively, of *Insr*. CB, cerebellum; BS, brain stem; TE, remainder of telencephalon and diencephalon; OB, olfactory bulb; M, muscle; L, liver; A, adipose tissue. ß-actin was used as a loading control. **B**–**F** Representative images of P21 retinal sections from WT mice. **B** Image of a retinal section immunostained for INSR (green). Nuclei are stained with DAPI (blue). **C**–**F** Magnified image of the OPL showing double (**C**–**E**) or triple (**F**) immunostaining for the indicated markers. In **F**, asterisks indicate cell bodies, arrows axons and arrow heads dendrites of horizontal cells. ONL Outer nuclear layer, OPL Outer plexiform layer, INL Inner nuclear layer, IPL Inner plexiform layer; NFL Nerve fiber layer. Scale bars: 90 μm (**B**), 45 μm (**C**–**E**) and 13 μm (**F**).

The insulin receptor gene (*Insr*) is expressed in mammals as two differentially spliced mRNA isoforms that differ by the presence of a small exon (exon 11) encoding 12 amino acids at the C-terminal of the α-subunit [31, 32]. The two isoforms (INSR-A and INSR-B) have distinct biochemical and functional properties and tissue distributions [32]. To first characterize the possible role of INSR signaling in the dystrophic retina, we evaluated the retinal expression of each isoform in both physiological and pathological conditions: retinal *Insr-a* and *Insr-b* expression was analyzed by reverse transcriptase polymerase chain reaction (RT-PCR) in WT mice and in the *Pde6b*^*rd10/rd10*^ (*rd10*) mouse model of RP [26]. Retinal RNA extracts were obtained at different stages from postnatal day 15 (P15) (i.e., before the appearance of evident retinal degeneration) up to P60 (after rod loss). The *Insr-a* isoform, which lacks exon 11, was the only isoform detected in both WT and *rd10* retinas at all stages analyzed, as well as in the four adult WT brain areas analyzed (cerebellum, brainstem, remainder of the telencephalon-diencephalon and olfactory bulb) (Fig. 1A). Conversely, key glucoregulatory tissues, such as the liver and adipose tissue, preferentially expressed the *Insr-b* isoform, which contains exon 11.

INSR tissue distribution was visualized by immunofluorescence using the anti-INSR β-subunit antibody C19 (see Table S1). The specificity of this antibody in neural tissue has been previously confirmed in *Insr*-knockout mice [33, 34]. We found that INSR was widely distributed in the WT retina, in accordance with its versatile role in the CNS. Interestingly, we observed prominent expression in the outer plexiform layer (OPL) and the retinal nerve fiber layer (NFL) (Fig. 1B). The OPL is a synaptic layer in which photoreceptor, horizontal, and bipolar cell axons and dendrites connect. Double immunostaining for INSR and calbindin, a marker of horizontal cells, revealed that INSR expression in the OPL was restricted to a subset of calbindin-positive fibers (Fig. 1C). By contrast, we observed no colocalization of INSR and PKC-α, which labels ON-bipolar cells (Fig. S2A). To determine the type (axon or dendrite) of INSR-positive horizontal cell processes, we performed immunostaining for neurofilament M (NF-M), which is selectively expressed by the axons [35, 36] (Fig. 1D, E). INSR expression in the OPL was restricted to NF-M-positive horizontal cell axons (Fig. 1D). NF-M-positive ganglion cell axons also showed robust INSR expression (Fig. S2B). Moreover, triple co-immunostaing for INSR, calbindin and NF-M confirmed the foremost location of INSR to horizontal cell axons (Fig. 1F). Horizontal cells receive rod and cone inputs separately; while their axon terminals receive inputs from rods, their dendrites collect input from cone pedicles [36–39]. Therefore, our results indicate preferential expression of INSR in the horizontal cell axons that synapse with rod spherules.

**Fig. 2:**
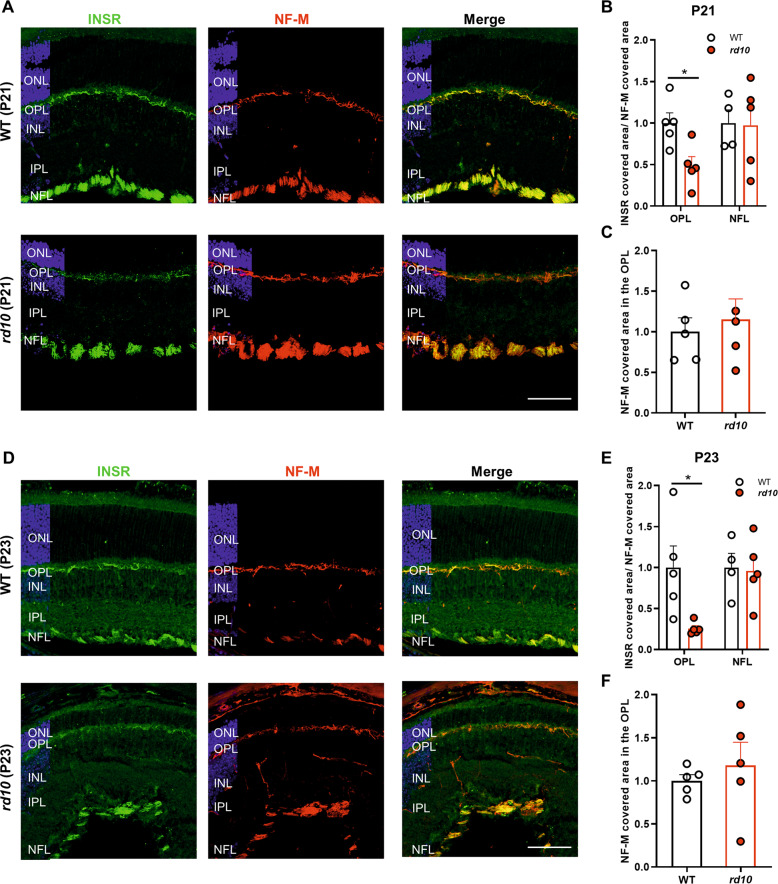
Downregulation of insulin receptor expression in the *rd10* mouse retina. **A**, **D** Representative images of P21 (**A**) and P23 (**D**) retinal sections from WT and *rd10* mice co-immunostained for INSR (green) and neurofilament-M (NF-M, red). Nuclei are stained with DAPI (blue). ONL Outer nuclear layer, OPL Outer plexiform layer, INL Inner nuclear layer, IPL Inner plexiform layer, NFL Nerve fiber layer. Scale bar: 66 μm. **B**, **E** Quantification of the area of INSR-positive staining in P21 (**B**) and P23 (**E**) WT and *rd10* retinal sections. The area corresponding to INSR immunostaining was normalized to that of neurofilament-M within the same region to correct for potential variations among retinal sections, and to the INSR/NF-M ratio in corresponding WT sections ( =1.0). **C**, **F** Quantification of the area of NF-M-positive staining in P21 (**C**) and P23 (**F**) WT and *rd10* retinal sections expressed relative to WT levels ( =1.0). Data are presented as the mean + SEM. *n* = 4–5 mice, 4 images per retina. *p ≤ 0.05 (unpaired-T test in **B** and **C** and unpaired-T test with Welch’s correction in **E** and **F**).

Next, we sought to characterize the pattern of INSR expression associated with retinal dystrophy. In early disease stages, before the onset of any significant disturbances in rod cells (P16), comparable global patterns of INSR immunostaining were observed in WT and *rd10* retinas (Fig. S3A, B). However, during the course of rod degeneration (P21–P23) we observed a selective and progressive decrease in INSR immunostaining in horizontal cell axons in the *rd10* retina (Fig. 2). This decrease was not due to the loss of horizontal cells, since similar numbers of calbindin-positive cells were observed in WT and *rd10* retinas at these ages (Fig. S4A, B), nor due to degeneration of horizontal cell axons, as evidenced by the preservation of NF-M immunostaining in the OPL (Fig. 2C, F). Moreover, INSR downregulation was specific to horizontal cell axons; we observed no significant changes in INSR immunostaining in ganglion cell axons (Fig. 2B, E).

**Fig. 3:**
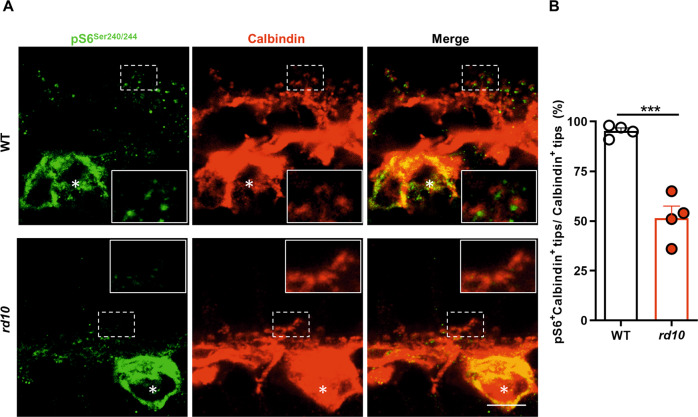
Downregulation of insulin receptor signaling in the *rd10* mouse retina. **A** Representative images of the OPL of P23 retinal sections from WT and *rd10* mice co-immunostained for pS6^Ser240/244^ (green) and calbindin (red). Asterisks indicate horizontal cell bodies. Insets show amplification (2.5X) of the indicated area. Scale bar: 6 μm. **B** Quantification of the number of horizontal terminal tips (calbindin^+^ puncta) with apposite pS6^Ser240/244^ labeling. Data are presented as the mean + SEM. *n* = 4 mice, 4 images. Over 200 calbindin^+^ tips were scored per retina. ***p ≤ 0.001 versus WT (unpaired T-test).

We next investigated whether the selective decrease in INSR expression in horizontal cell axons had consequences at the level of local INSR signaling. Of the multiple downstream effectors of INSR signaling, we specifically focused on ribosomal protein S6. A recent study [40] reported robust pS6^Ser240/244^ immunostaining in retinal horizontal and ganglion cells, where we observed prominent INSR expression. Immunostaining of WT retinas for pS6^Ser240/244^ corroborated the aforementioned labeling of horizontal and ganglion cell bodies (Fig. 3A, upper panels and Fig. S4C). Moreover, a closer examination of pS6^Ser240/244^ staining in the OPL revealed profuse punctate labeling in close apposition to the horizontal cell terminal tips (Fig. 3A, upper panels). Interestingly, pS6^Ser240/244^ immunostaining in the *rd10* retina revealed similar staining of horizontal cell bodies, but a dramatic decrease in punctate labeling (Fig. 3A, lower panels). Double immunostaining for pS6^Ser240/244^ and calbindin showed a decrease in the number of calbindin-positive tips due to retraction of horizontal terminal fibers caused by photoreceptor loss [3, 4]. Moreover, in the *rd10* retina half of the remaining horizontal cell terminal tips were devoid of pS6^Ser240/244^ labeling (Fig. 3A, lower panels, and Fig. 3B). This decrease was not due to lower levels of total S6 ribosomal protein, since similar staining for total S6 was observed in the OPL of WT and *rd10* retinas (Fig. S4D, E).

Taken together, our results indicate that in parallel with the degeneration of rod photoreceptors, the terminals of their post-synaptic horizontal cell partners undergo a putative process of insulin resistance, as evidenced by the decreases in INSR levels and pS6^Ser240/244^ signaling. This process may resemble the central and peripheral insulin resistance that occurs in brain neurodegenerative conditions and in type 2 diabetes [6, 41].

### Analysis of synaptic ultrastructure in the OPL of WT and rd10 mouse retinas

**Fig. 4:**
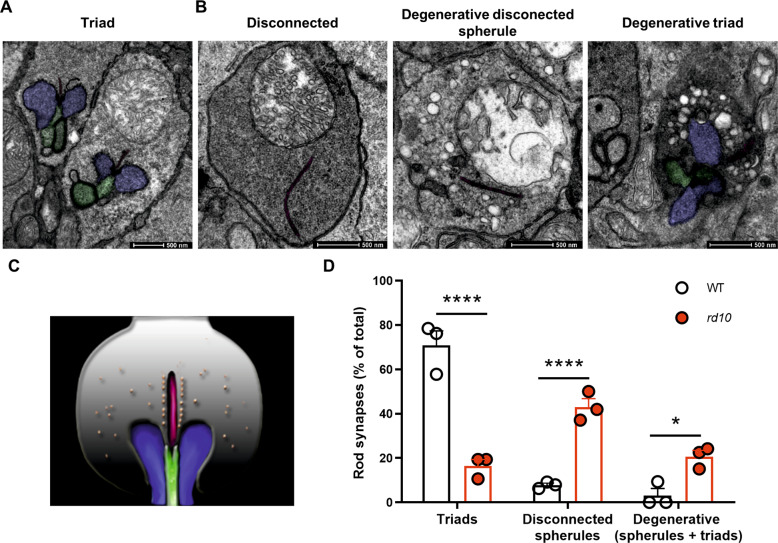
Ultrastructural analysis of rod photoreceptor synapses. **A**, **B** Representative electron microscopy images of P21 WT (**A**) and *rd10* (**B**) rod synapses. Synapses were categorized as follows: Triad, rod spherule with horizontal and bipolar invaginating postsynaptic terminals; Disconnected, viable rod spherule without postsynaptic elements; Degenerative disconnected spherule, rod spherule lacking postsynaptic profiles with characteristic signs of degeneration; Degenerative triad, rod spherule containing postsynaptic profiles but showing characteristic signs of degeneration. Scale bar: 500 nm. **C** Schematic diagram showing the structure of a rod triad synapse. The rod spherule (grey) consists of the synaptic ribbon (dark red), a scaffold structure containing presynaptic vesicles (orange circles). Horizontal cell postsynaptic terminals (blue) and bipolar dendrites (green) invaginate into the rod presynaptic terminal (spherule). **D** Quantification of the proportions of the different synaptic types. Results are expressed as the mean + SEM. *n* = 3 mice. Between 62 and 66 synapses were scored per mouse. ****p ≤ 0.0001, *p ≤ 0.05 (2-way ANOVA followed by Sidak’s multiple comparison test).

Given the marked downregulation of INSR expression and signaling in horizontal cell axons and terminal tips that accompanies rod degeneration, together with the role of INSR in synapse formation and maintenance [8, 14, 15], we next investigated whether changes in INSR expression coincided with alterations in synaptic structure in the OPL. Our analyses focused on rod synapses, since cone survival remains uncompromised in the early stages of degeneration in the *rd10* retina [42]. The OPL contains so-called triad synapses, formed by the presynaptic rod spherule, two lateral horizontal and one or two central bipolar postsynaptic terminals invaginating in close apposition to the synaptic ribbon (Fig. 4A, C). Using electron microscopy, we examined WT and *rd10* retinas in the early stages of degeneration (P21), when a large proportion of rod photoreceptor cells persist but signs of degeneration are also evident (Fig. S5). In WT retinas, most of the rod synapses (70%) corresponded to characteristic triad synapses (Fig. 4A, D). Conversely, in *rd10* retinas at the same timepoint, triad synapses accounted for less than 20% of all synapses (Fig. 4D). Moreover, in *rd10* retinas more than 40% of rod terminals lacked any postsynaptic element while in WT retinas this was observed in only 8% of rod terminals (Fig. 4B, D). Despite the absence of postsynaptic partners, these disconnected rod spherules had a viable appearance, as evidenced by intact mitochondria and electro-lucent content. In these disconnected spherules the synaptic ribbon, when present, was attached to an unusually flat cell membrane with no signs of any postsynaptic invagination (Fig. 4B). By contrast, the degenerative rod spherules (accounting for 20% and 3% of rod terminals in *rd10* and WT retinas, respectively) were highly vacuolated, with electro-dense content and aberrant mitochondria (Fig. 4B, D). Among the degenerated rod spherules, we observed both disconnected terminals and triad synapses (Fig. 4B), suggesting that disconnection is not a necessary step before degeneration. Furthermore, both genotypes showed similar numbers of dyads (18.65 ± 4.12% for WT vs 20.60 ± 3.01% for *rd10*), most likely due to the misalignment of one of the postsynaptic elements with the plane of section [43].

Immunostaining using specific markers for photoreceptor ribbon (Ribeye and Ctbp2) and horizontal (GluA2) and bipolar (mGluR6) postsynaptic terminal tips also revealed synaptic disconnection of rod photoreceptors in the *rd10* retina (Fig. S6A, B).

### Systemically-provided human proinsulin reaches the retina and exerts no metabolic effect

The results described above suggest a possible link between deficient INSR signaling, synaptic disconnection in rod photoreceptors, and the visual impairment characteristic of RP. To determine whether deficient INSR signaling is a disease-modifying factor in RP, and therefore a potential therapeutic target, we employed a gene therapy strategy to enhance INSR signaling. Proinsulin, the insulin precursor, is an INSR-A selective ligand [44] with a low metabolic profile, likely due to its poor affinity for the INSR-B isoform [32, 44]. We have previously demonstrated the neuroprotective potential of proinsulin during retinal development and degeneration [23–25, 45]. Given that treatment of RP in humans would entail long-term administration, we selected proinsulin to activate INSR-A, the predominant INSR isoform expressed in the retina (Fig. 1A).

**Fig. 5:**
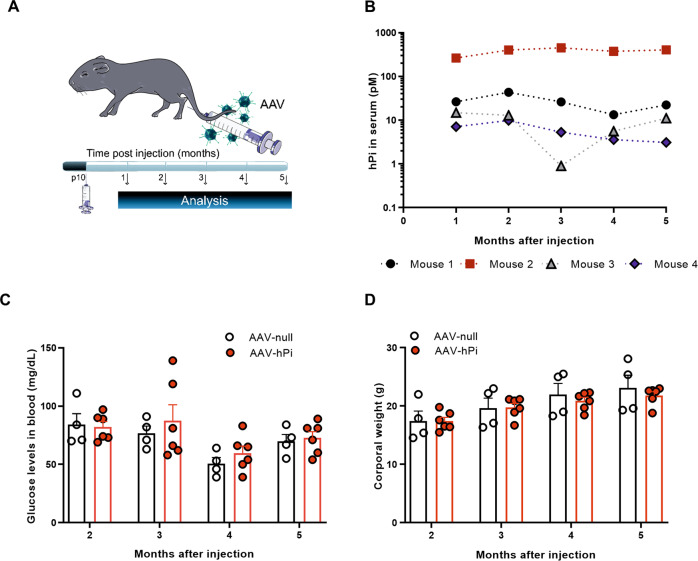
AAV-hPi treatment results in sustained human proinsulin production. **A** *rd10* mice received a single intramuscular injection of AAV-hPi or AAV-null at P10 and were analyzed at different timepoints post-injection. **B** Long-term monitoring of serum human proinsulin levels as determined by ELISA in individual *rd10* mice injected with AAV-hPi. Human proinsulin levels in AAV-null mice were under the detection threshold of the assay (0.5 pM). **C**, **D** Long-term monitoring of glucose levels (**C**) and body weight (**D**) in AAV-hPi or AAV-null injected *rd10* mice. Data are presented as the mean + SEM. *n* = 4 mice in **B** and 4–6 mice per group in **C** and **D**.

To achieve sustained production of Pi, we built upon our previous experience with a recombinant AAV2/1 expressing the human proinsulin (hPi) coding region (Fig. 5; AAV-hPi) [24, 27]. We first evaluated human proinsulin production following intramuscular administration of AAV-hPi. *rd10* mice received a single injection of AAV-hPi into the gastrocnemius muscle at P10 to induce human proinsulin production before the onset of degeneration (around P18). In AAV-hPi-treated *rd10* mice human proinsulin serum levels were uniformly sustained within each individual mouse for up to 5 months (the maximum follow-up period) (Fig. 5B). Moreover, human proinsulin was detected in whole eye and in retinal extracts as early as 1 week post-injection and up to 7 weeks post-injection (the maximum follow-up period) (Fig. S7A). Conversely, human proinsulin was not detected at any timepoint in serum samples, whole eye, or retinal extracts from mice injected with the control vector (AAV-null). Moreover, mature human insulin was absent in serum samples from AAV-hPi-treated WT mice, in agreement with our previous findings [27]. Therefore, human proinsulin produced from AAV-hPi remained mainly, if not completely, unprocessed. Importantly, we observed no differences in glycaemia or body weight in AAV-hPi- versus AAV-null-treated mice (Fig. 5C, D), confirming an absence of significant metabolic effects of systemic human proinsulin production, in line with previous studies [23, 24, 27].

### AAV-hPi treatment in rd10 mice preserves photoreceptors and photoreceptor synaptic connectivity

**Fig. 6:**
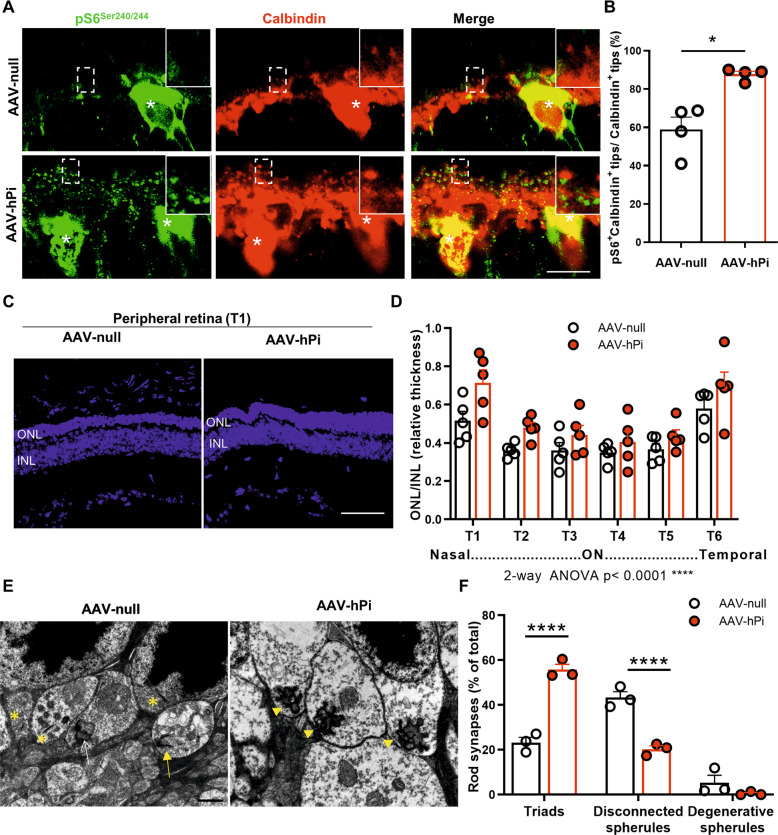
Effect of AAV-hPi administration on insulin receptor signaling and mouse retina structure. *rd10* mice received a single intramuscular injection of AAV-null or AAV-hPi at P12 and retinas were analyzed for pS6^Ser240/244^ expression (**A**, **B**), photoreceptor preservation (**C**, **D**) and synapse maintenance (**E**, **F**). **A** Representative images of the OPL in P21 retinal sections co-immunostained for pS6^Ser240/244^ (green) and calbindin (red). Asterisks indicate horizontal cell bodies. Insets show amplification (3X) of the indicated area. Scale bar: 11 μm. **B** Quantification of the number of horizontal terminal tips (calbindin^+^ puncta) with apposite pS6^Ser240/244^ labeling. Data are presented as the mean + SEM. *n* = 4 mice, 4 images. Over 200 calbindin^+^ tips per retina were analyzed. *p ≤ 0.05 (unpaired T-test with Welch’s correction). **C** Representative images of P30 retinal sections showing the T1 region from AAV-null- and AAV-hPi-treated *rd10* mice. Blue color corresponds to DAPI staining. ONL, outer nuclear layer; INL, inner nuclear layer. Scale bar: 70 μm. **D** ONL and INL thickness were measured in equatorial sections corresponding to 6 retinal areas, following a nasotemporal sequence (T1–T6 as defined in Fig. S1). Plot shows the mean + SEM. *n* = 5 mice, 3 sections per retina, 6 regions, 3 measurements per region. **E** Representative electron microscopy images of P30 AAV-null- and AAV-hPi-treated *rd10* mice. Arrow heads indicate triads, asterisks disconnected spherules, yellow arrow degenerative spherule and white arrow dyad. Scale bar: 1 μm. **F** Quantification of the proportions of the different synaptic types. Results are expressed as the mean + SEM. *n* = 3 mice. Between 58 and 86 synapses were scored per mouse. ****p ≤ 0.0001 (Sidak’s multiple comparisons test after 2-way ANOVA).

To determine the effect of proinsulin on the dystrophic retina, we first examined whether human proinsulin treatment could restore INSR signaling. AAV-hPi treated *rd10* retinas exhibited higher levels of punctate pS6^Ser240/244^ staining in the OPL than control (AAV-null-treated) *rd10* retinas (Fig. 6A, B). Quantification of the number of pS6^Ser240/244^-positive horizontal cell tips at P21 showed that human proinsulin treatment increased the proportion of calbindin-pS6^Ser240/244^-positive tips to near 90% (Fig. 6B), close to the proportion observed in WT retinas (Fig. 3B). By contrast, in control (AAV-null-treated) *rd10* retinas the proportion of pS6^Ser240/244^-positive horizontal cell tips remained in the range of 50–60% (Fig. 6B). The observed increment on pS6^Ser240/244^ was not due to higher amount of INSR or of total S6. Immunostaining and RT-qPCR analysis showed non-significant differences in INSR and S6 levels between the AAV-hPi and AAV-null *rd10* retinas (Fig. S8) indicating that human proinsulin action is via stimulation of INSR signaling. We also observed a greater abundance of total calbindin-positive tips in the AAV-hPi-treated than in the control retinas (Fig. 6A), most likely a consequence of photoreceptor preservation caused by human proinsulin treatment (Fig. 6C, D).

We next investigated whether long-term human proinsulin treatment exerted a neuroprotective effect. To this end, we injected *rd10* mice with AAV-null or AAV-hPi at P10–P12 and analyzed the corresponding retinas at P30, at which point most photoreceptor cells have been lost in this mouse model. Two distinct histological parameters were evaluated: photoreceptor cell preservation and synapse maintenance. AAV-hPi-treated *rd10* retinas showed modest but significant photoreceptor preservation, as determined by measuring the relative increase in thickness of the outer nuclear layer (ONL), an effect that was more evident in the nasal peripheral retina (T1; Fig. S1 and Fig. 6C, D). This observation correlated with decreased photoreceptor cell death (Fig. S9A, B). Immunostaning for rhodopsin and cone arrestin showed better structural preservation of photoreceptor outer segments than the control *rd10* mice (Figure S9C–F). In addition, the synaptic connectivity of photoreceptors was assessed by electron microscopy. The animals treated with human proinsulin presented higher proportion of connected rod spherules (triads) and decrease rate of spherules without postsynaptic elements (disconnected) than the control mice (Fig. 6E, F). These results were confirmed by immunostaining of rod-bipolar and rod-horizontal synapses (Fig. S6C–F). As expected, photoreceptor preservation in AAV-hPi-treated retinas led to an increase in the number of ribbons (Fig. S6C, D, lower panels). Moreover, human proinsulin reduced the proportion of disconnected postsynaptic terminals of rod cells [i.e. those lacking either a GluA2-positive horizontal postsynaptic terminal (Fig. S6C, E) or a mGluR6-positive bipolar postsynaptic terminal (Fig. S6D, F)] in agreement with the above electron microscopy results (Fig. 6E, F). These results reveal a novel effect of proinsulin: preservation of the synaptic connectivity of rod cells with their postsynaptic second-order neuronal partners. This observation suggests a second distinct role of human proinsulin that may be partially independent of its effects on photoreceptor cell survival revealed here (Fig. S9) as well as in our previous studies [23–25]. Moreover, the increase in the number of photoreceptor synapses induced by proinsulin treatment that we previously observed in the P23H rat model of RP [24] is most likely a consequence not only of the rescue of photoreceptor cells but also of the preservation of their synaptic contacts, as described here.

### AAV-hPi treatment in rd10 mice preserves visual function

**Fig. 7:**
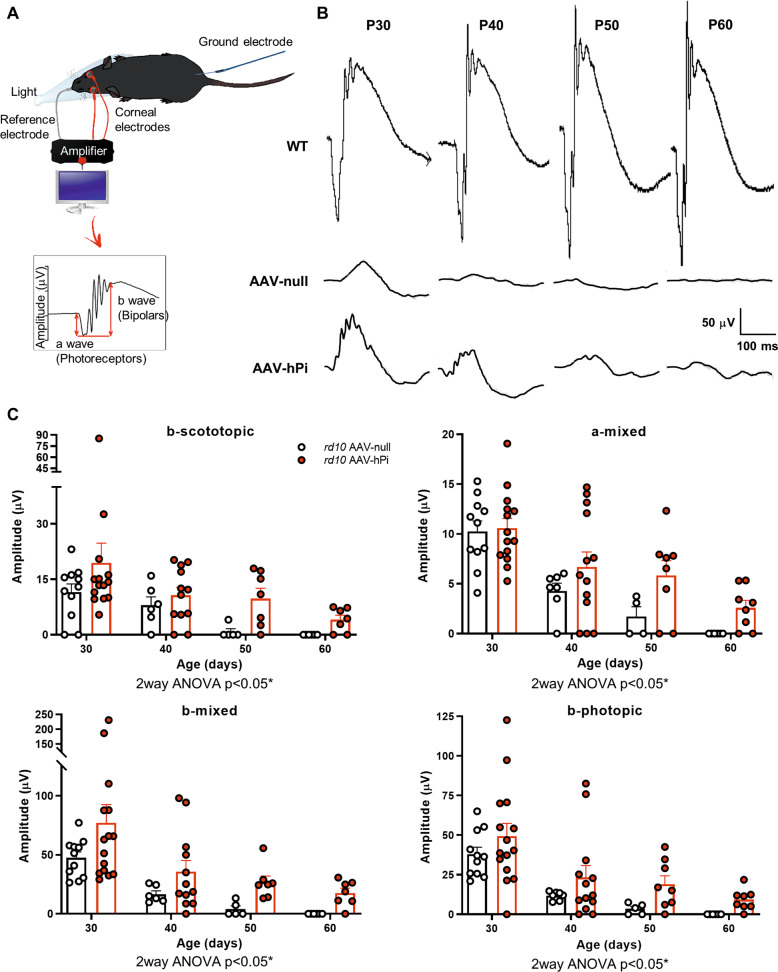
Effect of AAV-hPi administration on retinal response to light. *rd10* mice received a single intramuscular injection of AAV-null or AAV-hPi at P10. ERG recordings were performed at the indicated ages. **A** Schematic diagram depicting the ERG recording method. **B** Standard ERG representative trace recordings of the mixed responses obtained from 1 WT mouse, 1 AAV-null- and 1 AAV-hPi-treated over the course of the study (P30–P60) in response to a light stimulus of 1.5 cd·s/m^2^. Amplitudes of the a- and b-mixed waves are indicated on P30 trace recordings. **C** Graphs show averaged ERG wave amplitudes, plotted as a function of animal age. Amplitudes of the rod response (b-scotopic; light intensity = −2 log cd·s/m^2^) and rod and cone mixed response (a-mixed and b-mixed; light intensity = 2 log cd·s/m^2^) were recorded under scotopic conditions after overnight adaptation to darkness. Cone amplitudes (b-photopic; light intensity = 2 log cd·s/m^2^) were recorded after 5 minutes of light-adaptation (30 cd/m^2^ background light) under photopic conditions. Results are expressed as the mean + SEM. *n* = 4–13 mice. *p ≤ 0.05 (2-way ANOVA).

**Fig. 8:**
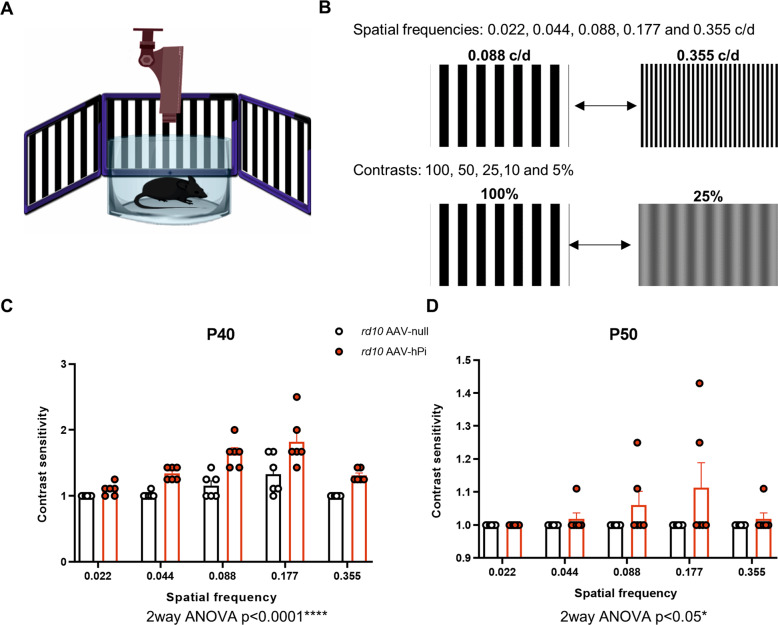
Effect of AAV-hPi administration on optokinetic response. *rd10* mice received a single intramuscular injection of AAV-null or AAV-hPi at P10. The optomotor test was performed at the indicated ages. **A**, **B** Schematic depicting the optomotor test (**A**); 5 different spatial frequencies were tested (0.022–0.355 cycles/degree) and the contrast of the moving bars was adjusted (from 100% to 5%) to determine contrast sensitivity (**B**). **C**, **D** Optokinetic responses recorded at the indicated ages in AAV-null- and AAV-hPi treated *rd10* mice. Contrast sensitivity is represented as a function of spatial frequency. Data are presented as the mean + SEM. *n* = 6 mice. P40 (****p ≤ 0.0001) and P50 (*p ≤ 0.05) (2-way ANOVA).

Finally, in AAV-hPi-treated *rd10* mice we assessed whether human proinsulin preserved visual function, which is the most clinically relevant outcome for a potential RP treatment. Electroretinographic (ERG) recordings were performed in dim and daylight conditions every 10 days between P30 and P60 to evaluate rod- and cone-mediated light responses. Mixed light responses of the WT animals experience small increase in ERG amplitudes from P30 to P60, however the light response of AAV-null animals was almost null at P50 (Fig. 7B). Of note, a remaining b-mixed wave was still observed in the P60 treated mouse (Fig. 7B). Analysis of different ERG waves showed that AAV-hPi-treated *rd10* mice displayed better defined and more prominent ERG waves than their AAV-null-treated counterparts (Fig. 7B). Waves corresponding to rods (b-scotopic wave), cones (b-photopic wave), and both photoreceptors (a-mixed and b-mixed waves) were of a significantly greater amplitude in AAV-hPi-treated than AAV-null-treated *rd10* mice (Fig. 7C). ERG recordings thus confirmed that human proinsulin treatment preserved visual function, consistent with the aforementioned preservation of photoreceptor cells and their synapses. Optokinetic testing further confirmed partial preservation of the light response in AAV-hPi-treated *rd10* retinas. In addition to measuring the retinal response to light, this visual behavior test evaluates the function of other components of the visual system, namely optic nerve transmission and visual integration in the brain [46]. Mice instinctively respond to rotating vertical bars with characteristic movement of their heads in the same direction as the rotation of the bar (Fig. 8A, B). AAV-hPi-treated *rd10* mice showed greater contrast sensitivity than AAV-null-treated counterparts at P40 (Fig. 8C). Moreover, at P50, age at which all control mice have lost the optokinetic response, two out of six proinsulin-treated *rd10* showed significant contrast sensitivity (Fig. 8D). Together, our results support the disease-modifying potential of proinsulin, which can prolong visual function in an animal model of RP and therefore constitutes a worthwhile candidate therapy for retinal dystrophies.

## Discussion

This study describes the downregulation of retinal INSR levels and local signaling during the early stages of retinal neurodegeneration in the *rd10* mouse model of RP, together with concomitant disruption of photoreceptor triad synapses. We provide proof of concept of the neuroprotective effect of INSR stimulation with the insulin precursor proinsulin, which is selective for the INSR-A isoform found in the retina. Gene therapy using an AAV induced sustained production of circulating proinsulin, which reached the retina, restored local INSR signaling, and exerted neuroprotective effects on retinal structure and visual function, without affecting peripheral metabolic parameters. Proinsulin treatment attenuated both photoreceptor cell loss and synaptic disconnection, and prolonged visual function, highlighting the potential of proinsulin as a candidate therapy for RP.

Studies over the past two decades have broadened the scope of INSR activity far beyond the peripheral metabolic role initially ascribed to this receptor. The versatility of this receptor is also implied by its widespread expression in the CNS. At the neuronal level, INSR has been implicated in synaptic plasticity, dendritic outgrowth, and cell survival [8, 14, 15]. The *Insr-a* splice variant, which is predominantly expressed in different areas of the brain as well as the retina, is the most ancient and promiscuous isoform, and participates in insulin, proinsulin, and IGF-II signaling [31, 44]. Interestingly, many of the signaling pathways in which INSR-A is involved are similar to those mediated by growth factor receptors, and in some ways this isoform resembles the ancestral INSR expressed in invertebrates and low vertebrates [7, 47, 48]. The present findings confirm the previously described wide distribution of INSR in the WT retina [49, 50]. However, we also describe for the first time more intense INSR immunostaining in horizontal and ganglion cell axons than in other retinal structures. Interestingly, analysis of INSR expression during retinal degeneration in the *rd10* mouse revealed local downregulation of INSR, specifically in horizontal cell axons. This was accompanied by a decrease in the phosphorylation of ribosomal protein S6 in horizontal cell terminals, indicating impaired INSR signaling. Notably, horizontal cell axon terminals receive input from the rod photoreceptors, the main targets of degeneration in RP. A recent study by Agostinone et al. [40] reported specific decreases in pS6^Ser240/244^ in ganglion cells following transection of their axons, but no alterations in pS6^Ser240/244^ in horizontal cells. In the present study, we observed selective downregulation of INSR expression and impaired signaling in the horizontal cells with which the damaged rod photoreceptors synapse. It remains unclear whether this finding is mechanistically linked to the decreases in pS6^Ser240/244^ described by Agostinone et al. [40]. However, our results are consistent with the proposals by other authors that INSR signaling may vary in a neuronal activity-dependent manner [8, 51, 52], and suggest that INSR expression and signaling in horizontal cells may depend on rod input.

Interestingly, in *rd10* retinas INSR downregulation and consequent impairment of INSR signaling coincided with the presence of disconnected but otherwise apparently viable rod terminals. Alterations in retinal synaptic circuitry as part of retinal remodeling during photoreceptor degeneration have been acknowledged for more than two decades [3, 53–55]. De-afferentiation of second-order neurons has typically been described in advanced stages of degeneration as a consequence of photoreceptor loss [3, 53–55]. However, here we describe early disconnection events affecting up to 50% of apparently viable rod spherules during the initial stages of degeneration. Although further studies will be required to clarify the consequences of this disconnection, it does not appear to necessarily precede rod death, as we detected degenerative triads containing the presynaptic rod spherule and horizontal and bipolar terminals. These observations have direct implications for the development of neuroprotective therapies and could open a new line of research into interventions aimed at preserving rod connectivity.

Our findings demonstrate the potential of proinsulin as a synaptoprotective factor. In *rd10* mice treated with AAV-hPi, systemically produced proinsulin reached the retina and restored INSR signaling, as determined by measuring pS6^Ser240/244^ levels. Concomitantly, proinsulin treatment reduced the number of disconnected rod presynaptic terminals, suggesting a role of INSR signaling in photoreceptor synaptic connectivity. Our results are in line with those of the seminal study by Chiu et al. 2008 [8], who used optic tectal neurons in living *Xenopus* tadpoles to demonstrate that INSR signaling maintains both synaptic contacts and the branches on which they lie. Moreover, in the aforementioned study by Agostinone et al. [40] stimulation of INSR signaling with insulin promoted regeneration of the dendritic arbors of retinal ganglion cells after axonal injury.

Deficient local INSR signaling associated with RP may be a general feature of neurodegenerative diseases, irrespective of peripheral insulin resistance [reviewed in [6]]. Several studies have described decreased INSR expression and/or attenuation of the activation states of INSR signaling molecules in affected brain regions in patients with Alzheimer’s [17–19] and Parkinson’s [56–58] diseases. We show that pharmacological stimulation of INSR signaling with Pi has a disease-modifying effect over the course of retinal degeneration, in line with the beneficial effects of INSR stimulation with insulin reported in other neurodegenerative diseases of the brain and retina [6, 40, 41]. Moreover, the widespread expression of INSR in the retina suggests that synaptic maintenance promoted by INSR signaling is only one of several retinal processes regulated by INSR. Indeed, we and others have demonstrated the neuroprotective effects of INSR signaling molecules on photoreceptor survival [28, 59, 60].

Our results provide a novel mechanism to account for our previous descriptions of the neuroprotective effects of proinsulin on retinal dystrophy [23–25] and cognitive impairment [27], and further support the validity of proinsulin-mediated stimulation of INSR as a candidate therapy for neurodegenerative conditions of the CNS.

## Supplementary information

Supporting information

Supplementary Table 1

Supplementary Figure S1

Supplementary Figure S2

Supplementary Figure S3

Supplementary Figure S4

Supplementary Figure S5

Supplementary Figure S6

Supplementary Figure S7

Supplementary Figure S8

Supplementary Figure S9

Original Data - 1A

Reproducibility checklist

## Data Availability

All analyzed datasets are included in the manuscript and SI Appendix.

## References

[1] Collaborators GBDN (2019). Global, regional, and national burden of neurological disorders, 1990-2016: a systematic analysis for the Global Burden of Disease Study 2016. Lancet Neurol.

[2] de La Rosa EJ, Hernandez-Sanchez C. CNS Targets for the Treatment of Retinal Dystrophies: A Win–Win Strategy. Therapies for retinal degeneration: Targeting common processes. de la Rosa EJ, Cotter TG Editors. Royal Society of Chemistry; 2019. p. 277.

[3] Pfeiffer RL, Marc RE, Jones BW (2020). Persistent remodeling and neurodegeneration in late-stage retinal degeneration. Prog Retin Eye Res.

[4] Cuenca N, Fernandez-Sanchez L, Campello L, Maneu V, De la Villa P, Lax P (2014). Cellular responses following retinal injuries and therapeutic approaches for neurodegenerative diseases. Prog Retin Eye Res.

[5] Russell S, Bennett J, Wellman JA, Chung DC, Yu ZF, Tillman A (2017). Efficacy and safety of voretigene neparvovec (AAV2-hRPE65v2) in patients with RPE65-mediated inherited retinal dystrophy: A randomised, controlled, open-label, phase 3 trial. Lancet.

[6] Arnold SE, Arvanitakis Z, Macauley-Rambach SL, Koenig AM, Wang HY, Ahima RS (2018). Brain insulin resistance in type 2 diabetes and Alzheimer disease: Concepts and conundrums. Nat Rev Neurol.

[7] Banks WA, Owen JB, Erickson MA (2012). Insulin in the brain: There and back again. Pharm Ther.

[8] Chiu SL, Cline HT (2010). Insulin receptor signaling in the development of neuronal structure and function. Neural Dev.

[9] de la Rosa EJ, Bondy CA, Hernandez-Sanchez C, Wu X, Zhou J, Lopez-Carranza A (1994). Insulin and insulin-like growth factor system components gene expression in the chicken retina from early neurogenesis until late development and their effect on neuroepithelial cells. Eur J Neurosci.

[10] Havrankova J, Roth J, Brownstein M (1978). Insulin receptors are widely distributed in the central nervous system of the rat. Nature.

[11] Marks JL, Porte D, Stahl WL, Baskin DG (1990). Localization of insulin receptor mRNA in rat brain by in situ hybridization. Endocrinology.

[12] Rodrigues M, Waldbillig RJ, Rajagopalan S, Hackett J, LeRoith D, Chader GJ (1988). Retinal insulin receptors: Localization using a polyclonal anti-insulin receptor antibody. Brain Res.

[13] Unger J, McNeill TH, Moxley RT, White M, Moss A, Livingston JN (1989). Distribution of insulin receptor-like immunoreactivity in the rat forebrain. Neuroscience.

[14] Gralle M (2017). The neuronal insulin receptor in its environment. J Neurochem.

[15] Lee CC, Huang CC, Hsu KS (2011). Insulin promotes dendritic spine and synapse formation by the PI3K/Akt/mTOR and Rac1 signaling pathways. Neuropharmacology.

[16] Moloney AM, Griffin RJ, Timmons S, O’Connor R, Ravid R, O’Neill C (2010). Defects in IGF-1 receptor, insulin receptor and IRS-1/2 in Alzheimer’s disease indicate possible resistance to IGF-1 and insulin signalling. Neurobiol Aging.

[17] Rivera EJ, Goldin A, Fulmer N, Tavares R, Wands JR, de la Monte SM (2005). Insulin and insulin-like growth factor expression and function deteriorate with progression of Alzheimer’s disease: link to brain reductions in acetylcholine. J Alzheimers Dis.

[18] Steen E, Terry BM, Rivera EJ, Cannon JL, Neely TR, Tavares R (2005). Impaired insulin and insulin-like growth factor expression and signaling mechanisms in Alzheimer’s disease-is this type 3 diabetes?. J Alzheimers Dis.

[19] Talbot K, Wang HY, Kazi H, Han LY, Bakshi KP, Stucky A (2012). Demonstrated brain insulin resistance in Alzheimer’s disease patients is associated with IGF-1 resistance, IRS-1 dysregulation, and cognitive decline. J Clin Invest.

[20] Chapman CD, Schioth HB, Grillo CA, Benedict C (2018). Intranasal insulin in Alzheimer’s disease: Food for thought. Neuropharmacology.

[21] Hernandez-Sanchez C, Lopez-Carranza A, Alarcon C, de La Rosa EJ, de Pablo F (1995). Autocrine/paracrine role of insulin-related growth factors in neurogenesis: local expression and effects on cell proliferation and differentiation in retina. Proc Natl Acad Sci USA.

[22] Valenciano AI, Corrochano S, de Pablo F, de la Villa P, de la Rosa EJ (2006). Proinsulin/insulin is synthesized locally and prevents caspase- and cathepsin-mediated cell death in the embryonic mouse retina. J Neurochem.

[23] Corrochano S, Barhoum R, Boya P, Arroba AI, Rodriguez-Muela N, Gomez-Vicente V (2008). Attenuation of vision loss and delay in apoptosis of photoreceptors induced by proinsulin in a mouse model of retinitis pigmentosa. Invest Ophthalmol Vis Sci.

[24] Fernandez-Sanchez L, Lax P, Isiegas C, Ayuso E, Ruiz JM, de la Villa P (2012). Proinsulin slows retinal degeneration and vision loss in the P23H rat model of retinitis pigmentosa. Hum Gene Ther.

[25] Isiegas C, Marinich-Madzarevich JA, Marchena M, Ruiz JM, Cano MJ, de la Villa P (2016). Intravitreal injection of proinsulin-loaded microspheres delays photoreceptor cell death and vision loss in the rd10 mouse model of retinitis pigmentosa. Invest Ophthalmol Vis Sci.

[26] Chang B, Hawes NL, Pardue MT, German AM, Hurd RE, Davisson MT (2007). Two mouse retinal degenerations caused by missense mutations in the beta-subunit of rod cGMP phosphodiesterase gene. Vis Res.

[27] Corpas R, Hernandez-Pinto AM, Porquet D, Hernandez-Sanchez C, Bosch F, Ortega-Aznar A (2017). Proinsulin protects against age-related cognitive loss through anti-inflammatory convergent pathways. Neuropharmacology.

[28] Sanchez-Cruz A, Villarejo-Zori B, Marchena M, Zaldivar-Diez J, Palomo V, Gil C (2018). Modulation of GSK-3 provides cellular and functional neuroprotection in the rd10 mouse model of retinitis pigmentosa. Mol Neurodegener.

[29] Sánchez-Cruz A, Méndez AC, Lizasoain I, de la Villa P, de la Rosa EJ, Hernández-Sánchez C (2021). Tlr2 gene deletion delays retinal degeneration in two genetically distinct mouse models of retinitis pigmentosa. Int J Mol Sci.

[30] Prusky GT, Alam NM, Beekman S, Douglas RM (2004). Rapid quantification of adult and developing mouse spatial vision using a virtual optomotor system. Invest Ophthalmol Vis Sci.

[31] Hernandez-Sanchez C, Mansilla A, de Pablo F, Zardoya R (2008). Evolution of the insulin receptor family and receptor isoform expression in vertebrates. Mol Biol Evol.

[32] Belfiore A, Malaguarnera R, Vella V, Lawrence MC, Sciacca L, Frasca F (2017). Insulin receptor isoforms in physiology and disease: An updated view. Endocr Rev.

[33] Bruning JC, Gautam D, Burks DJ, Gillette J, Schubert M, Orban PC (2000). Role of brain insulin receptor in control of body weight and reproduction. Science.

[34] Dixon-Salazar TJ, Fourgeaud L, Tyler CM, Poole JR, Park JJ, Boulanger LM (2014). MHC class I limits hippocampal synapse density by inhibiting neuronal insulin receptor signaling. J Neurosci.

[35] Peichl L, Gonzalez-Soriano J (1993). Unexpected presence of neurofilaments in axon-bearing horizontal cells of the mammalian retina. J Neurosci.

[36] Peichl L, Gonzalez-Soriano J (1994). Morphological types of horizontal cell in rodent retinae: a comparison of rat, mouse, gerbil, and guinea pig. Vis Neurosci.

[37] Feigenspan A, Babai N (2015). Functional properties of spontaneous excitatory currents and encoding of light/dark transitions in horizontal cells of the mouse retina. Eur J Neurosci.

[38] Kolb H (1970). Organization of the outer plexiform layer of the primate retina: Electron microscopy of Golgi-impregnated cells. Philos Trans R Soc Lond B Biol Sci.

[39] Kolb H (1974). The connections between horizontal cells and photoreceptors in the retina of the cat: Electron microscopy of Golgi preparations. J Comp Neurol.

[40] Agostinone J, Alarcon-Martinez L, Gamlin C, Yu WQ, Wong ROL, Di Polo A (2018). Insulin signalling promotes dendrite and synapse regeneration and restores circuit function after axonal injury. Brain.

[41] Holscher C (2020). Brain insulin resistance: Role in neurodegenerative disease and potential for targeting. Expert Opin Investig Drugs.

[42] Zhao L, Zabel MK, Wang X, Ma W, Shah P, Fariss RN (2015). Microglial phagocytosis of living photoreceptors contributes to inherited retinal degeneration. EMBO Mol Med.

[43] Wang T, Pahlberg J, Cafaro J, Frederiksen R, Cooper AJ, Sampath AP (2019). Activation of rod input in a model of retinal degeneration reverses retinal remodeling and induces formation of functional synapses and recovery of visual signaling in the adult retina. J Neurosci.

[44] Malaguarnera R, Sacco A, Voci C, Pandini G, Vigneri R, Belfiore A (2012). Proinsulin binds with high affinity the insulin receptor isoform A and predominantly activates the mitogenic pathway. Endocrinology.

[45] Hernandez-Sanchez C, Mansilla A, de la Rosa EJ, de Pablo F (2006). Proinsulin in development: New roles for an ancient prohormone. Diabetologia.

[46] Abdeljalil J, Hamid M, Abdel-Mouttalib O, Stephane R, Raymond R, Johan A (2005). The optomotor response: A robust first-line visual screening method for mice. Vis Res.

[47] Belfiore A, Frasca F, Pandini G, Sciacca L, Vigneri R (2009). Insulin receptor isoforms and insulin receptor/insulin-like growth factor receptor hybrids in physiology and disease. Endocr Rev.

[48] Chan SJ, Steiner DF (2000). Insulin Through the Ages: Phylogeny of a Growth Promoting and Metabolic Regulatory Hormone 1. Am Zool.

[49] Gosbell AD, Favilla I, Baxter KM, Jablonski P (2000). Insulin receptor and insulin receptor substrate-I in rat retinae. Clin Exp Ophthalmol.

[50] Rajala RV, Wiskur B, Tanito M, Callegan M, Rajala A (2009). Diabetes reduces autophosphorylation of retinal insulin receptor and increases protein-tyrosine phosphatase-1B activity. Invest Ophthalmol Vis Sci.

[51] Clarke DW, Mudd L, Boyd FT, Fields M, Raizada MK (1986). Insulin is released from rat brain neuronal cells in culture. J Neurochem.

[52] Hori K, Yasuda H, Konno D, Maruoka H, Tsumoto T, Sobue K (2005). NMDA receptor-dependent synaptic translocation of insulin receptor substrate p53 via protein kinase C signaling. J Neurosci.

[53] Jones BW, Kondo M, Terasaki H, Lin Y, McCall M, Marc RE (2012). Retinal remodeling. Jpn J Ophthalmol.

[54] Lewis GP, Linberg KA, Fisher SK (1998). Neurite outgrowth from bipolar and horizontal cells after experimental retinal detachment. Invest Ophthalmol Vis Sci.

[55] Strettoi E, Pignatelli V, Rossi C, Porciatti V, Falsini B (2003). Remodeling of second-order neurons in the retina of rd/rd mutant mice. Vis Res.

[56] Moroo I, Yamada T, Makino H, Tooyama I, McGeer PL, McGeer EG (1994). Loss of insulin receptor immunoreactivity from the substantia nigra pars compacta neurons in Parkinson’s disease. Acta Neuropathol.

[57] Takahashi M, Yamada T, Tooyama I, Moroo I, Kimura H, Yamamoto T (1996). Insulin receptor mRNA in the substantia nigra in Parkinson’s disease. Neurosci Lett.

[58] Timmons S, Coakley MF, Moloney AM, ON C (2009). Akt signal transduction dysfunction in Parkinson’s disease. Neurosci Lett.

[59] Punzo C, Kornacker K, Cepko CL (2009). Stimulation of the insulin/mTOR pathway delays cone death in a mouse model of retinitis pigmentosa. Nat Neurosci.

[60] Rajala A, Tanito M, Le YZ, Kahn CR, Rajala RV (2008). Loss of neuroprotective survival signal in mice lacking insulin receptor gene in rod photoreceptor cells. J Biol Chem.

