# Supplementary material for: Insulin receptor activation by proinsulin preserves synapses and vision in retinitis pigmentosa
Source: Cell Death Dis. 2022 Apr 20;13(4):383. doi: 10.1038/s41419-022-04839-0 (PMC9021205; doi:10.1038/s41419-022-04839-0)
Supplement: Supplementary file 1 — Supporting information [file 41419_2022_4839_MOESM1_ESM.docx]

**Supplementary information for:**

Insulin receptor activation by proinsulin preserves synapses and vision in retinitis pigmentosa.

Alonso Sánchez-Cruz, Alberto Hernández-Pinto, Concepción Lillo, Carolina Isiegas, Miguel Marchena, Ignacio Lizasoain, Fátima Bosch, Pedro de la Villa, Catalina Hernández-Sánchez and Enrique J. de la Rosa.

Catalina Hernández-Sánchez and Enrique J. de la Rosa

Email: [chernandez@cib.csic.es](mailto:chernandez@cib.csic.es); [ejdelarosa@cib.csic.es](file:///C:\Users\Cati%20Hernández\Downloads\ejdelarosa@cib.csic.es)

**Figure S1. Schematic depicting retinal sections.** The 6 retinal zones in which quantification was performed (T1, T2, T3, T4, T5 and T6) are indicated. ON, optic nerve.

**Figure S2. Analysis of insulin receptor expression.** Representative retinal sections collected from WT mice at P21. **A, B**. Magnified image showing the OPL (A) and NFL (B) after co-immunostaining for the indicated markers. Insets in A show magnification (1.5X) of the indicated area. Nuclei are stained with DAPI (blue). OPL, outer plexiform layer; NFL, nerve fiber layer. Scale bar: 16 μm (A) and 21 μm (B).

**Figure S3. Analysis of insulin receptor expression in WT and *rd10* mouse retinas at P16. A.** Representative images of P16 retinal sections from WT and *rd10* mice co-immunostained for INSR (green) and neurofilament-M (NF-M, red). Nuclei are stained with DAPI (blue). ONL, outer nuclear layer; OPL, outer plexiform layer; INL, inner nuclear layer; IPL, inner plexiform layer; NFL, nerve fiber layer. Scale bar: 58 μm. **B.** Quantification of the area of INSR-positive immunostaining at P16 in retinal sections from WT and *rd10* mice. The area of INSR-positive immunostaining was normalized to that of neurofilament-staining of the same region to correct for potential variation among retinal sections, and to the INSR/NF-M ratio in WT sections (=1.0). **C.** Quantification of the area of NF-M-positive staining in P16 WT and *rd10* retinal sections expressed relative to WT levels (=1.0). Data are presented as the mean + SEM. n=3-4 mice, 4 images per retina.

**Figure S4. Comparison of the number of horizontal cells, pS6^Ser240/244^ and S6 staining in WT and *rd10* mouse retinas. A, C.** Representative images of P23 whole retinal sections (A) and GCL (C) from WT and *rd10* mice, immunostained for calbindin to label horizontal cells (A, red) or pS6^Ser240/244^ (C, green). Nuclei are stained with DAPI (blue). **B.** The number of calbindin^+^ cells was scored in equatorial sections in the central regions of the retina (T3–T4; see Methods and Figure S1). Plots show the mean + SEM. n=5 mice, 10 images per retina. **D.** Representative images of the OPL of P23 WT and *rd10* retinas, immunostained for S6 (green) and calbindin (red). **E.** Quantificacion of S6 staining in the OPL in WT and *rd10* retinas expressed relative to WT levels (=1.0). Plots show the mean +SEM. n=4 mice, 4 images per retina, 4 measurements per image (see Methods). ONL, outer nuclear layer; OPL, outer plexiform layer; INL, inner nuclear layer; GCL, ganglion cell layer. Scale bars: 38 μm (A) and 41 μm (B) and 11 μm (D).

**Figure S5. Representative semi-thin retinal sections. A**. Semi-thin retinal sections collected at P21 from WT and *rd10* mice were prepared for electron microscopy analysis (shown in Fig. 4). Scale bar: 50 μm. **B**. Magnification of the indicated region. Black arrows indicate photoreceptor presynaptic terminals. White asterisks indicate degenerating photoreceptors, as evidenced by the condensed nucleus. ONL, outer nuclear layer; OPL, outer plexiform layer; INL, inner nuclear layer; IPL, inner plexiform layer. Scale bar: 100 μm.

**Figure S6.** **Representative 3D reconstructions of synapses in the OPL.** **A-D.** Retinal sections were co-immunostained for Ribeye (ribbon at presynaptic terminal, red) and the glutamate receptor subunit GluA2 (horizontal postsynaptic terminal, green) (A, C), or for Ctbp2 (ribbon at presynaptic terminal, red) and the glutamate receptor mGluR6 (bipolar postsynaptic terminal, green) (B, D). **A, B.** OPL of P23 WT and *rd10*. **C, D.** OPL of *rd10* mice that received a single intramuscular injection of AAV-null or AAV-hPi at P12 and were analyzed at P30. Arrowheads indicate ribbons (rod presynaptic terminals) without a post-synaptic partner. OPL, outer plexiform layer. Scale bars: 6 μm (A, B) and 3 μm (C, D). **E, F.** Quantification of the number of disconnected rod presynaptic terminals. Percentage of ribbons without associated GluA2 (C) or mGluR6 (D) punctate staining. Plots show the mean + SEM. *n* = 5 mice. Over 200 ribbons were analyzed per retina. **p ≤0.01, *p ≤ 0.05 (unpaired T-test).

**Figure S7. Determination of human proinsulin levels after AAV-hPi treatment.** *rd10* mice received a single intramuscular injection of AAV-hPi or AAV-null at P10. **A, B**. ELISA was performed to measure hPi in retinal and eye extracts (A) and in serum (B) at the indicated times. Graphs show hPi levels in individual *rd10* mice injected with AAV-hPi. Human proinsulin levels were relativized to mg of protein of the retinal or eye extract (A). Human proinsulin levels in the AAV-null mice were under the detection threshold of the assay (0.5 pM). Data are presented as the mean + SEM. n=3 mice. *p ≤ 0.05 (1-way ANOVA with Dunnett’s multiple comparison test).

**Figure S8. INSR and total S6 expression in *rd10* mouse retina after AAV-hPi treatment.** *rd10* mice received a single intramuscular injection of either AAV-null or AAV-hPi at P12 and were analyzed at P21 for INSR and S6 expression. **A, C.** Representative images of retinal sections of AAV-null or AAV-hPi treated *rd10* mouse co-immunostained for INSR (green) and NF-M (red) (A) or S6 (green) and calbindin (red) (C). **B.** Quantification of INSR covered area normalized to NF-M and relativized to that of AAV-null injected *rd10* mouse (=1). n=4 mice, 4 images per retina. **D.** Quantification of the S6 staining in the OPL of AAV-null or AAV-hPi treated *rd10* mouse, relativized to that of AAV-null injected *rd10* mouse (=1). Dots represent individual mice, while bars show the mean of each group (+SEM). n=4 mice, 4 images per retina and 4 measurements per image (see Methods). **E, F.** Insulin receptor (*Insr,* E) and S6 ribosomal protein (*Rps6*, F) gene expression was analyzed by RT-qPCR in *rd10* mouse retinas after AAV-null or AAV-hPi treatment. The levels of the transcripts were normalized to the *Tbp* RNA and relativized to AAV-null levels injected *rd10* mouse (=1). n=4 mice. Plots show the mean +SEM.

**Figure S9.** **Effect of AAV-hPi administration on photoreceptor preservation.** *rd10* mice received a single intramuscular injection of AAV-null or AAV-hPi at P12 and retinas were subjected to TUNEL assay and analyzed for rhodopsin and cone-arrestin expression at P30**. A.** Representative retinal images of AAV-null- and AAV-hPi-treated *rd10* mice stained with TUNEL assay. **B.** The number of TUNEL^+^ nuclei in the ONL were quantified in the whole retinal section. n=3 mice, 3 retinal sections. **C and E.** Representative retinal images of AAV-null- and AAV-hPi-treated *rd10* mice stained for rhodopsin (C) and cone-arrestin (E). **D, F.** The length of rod (D) or cone (F) OS was measured in the peripheral retinas of AAV-null- and AAV-hPi-treated *rd10* mice. n=5 mice, 6 images per retina and 3 measurements per image (see Methods). Plots show the mean +SEM. *p ≤ 0.05 (unpaired T-test).
