# Supplementary figures and images for: Insulin receptor activation by proinsulin preserves synapses and vision in retinitis pigmentosa
Source: Cell Death Dis. 2022 Apr 20;13(4):383. doi: 10.1038/s41419-022-04839-0 (PMC9021205; doi:10.1038/s41419-022-04839-0)

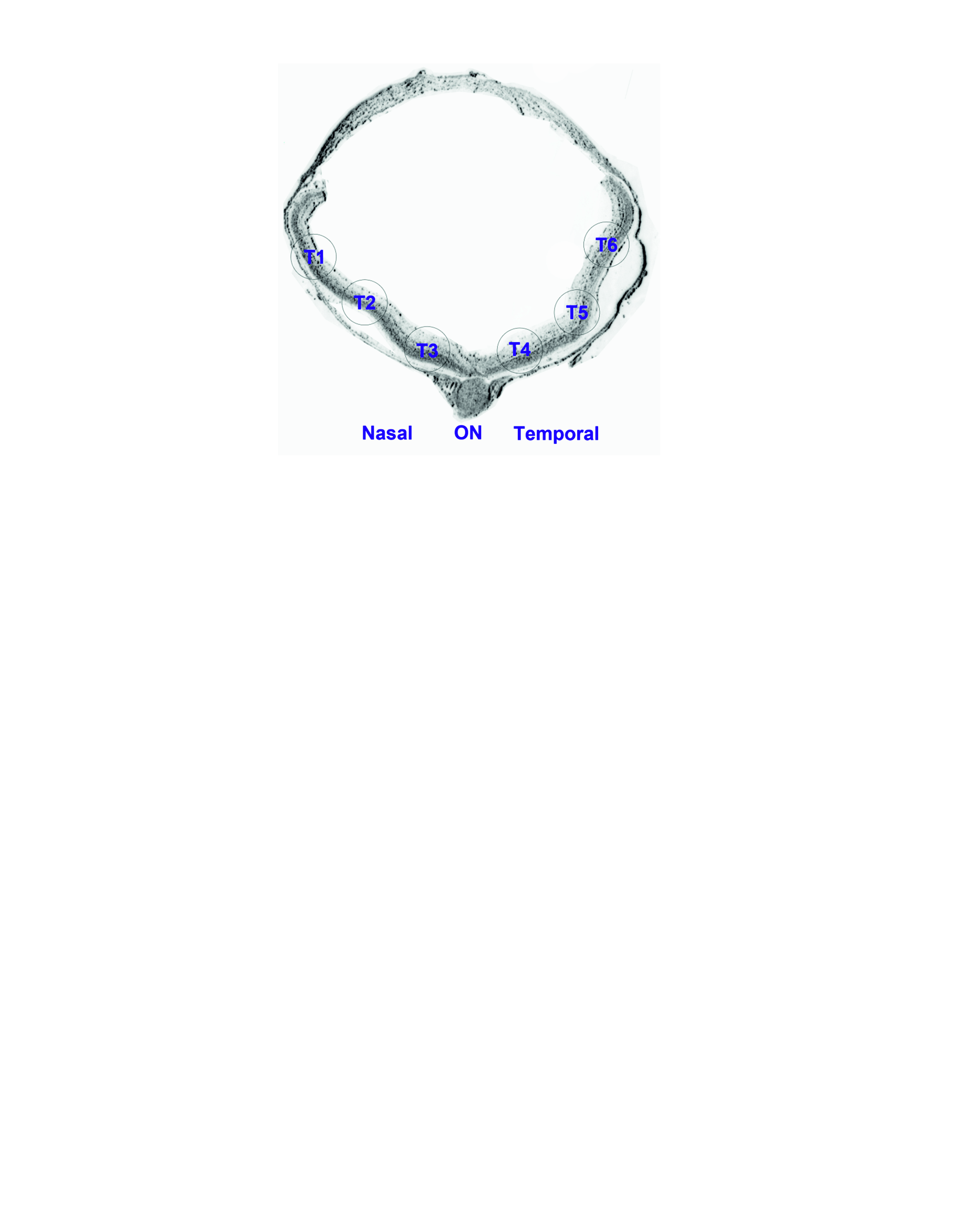

Supplement: Supplementary file 3 — Supplementary Figure S1 [file 41419_2022_4839_MOESM3_ESM.tif]

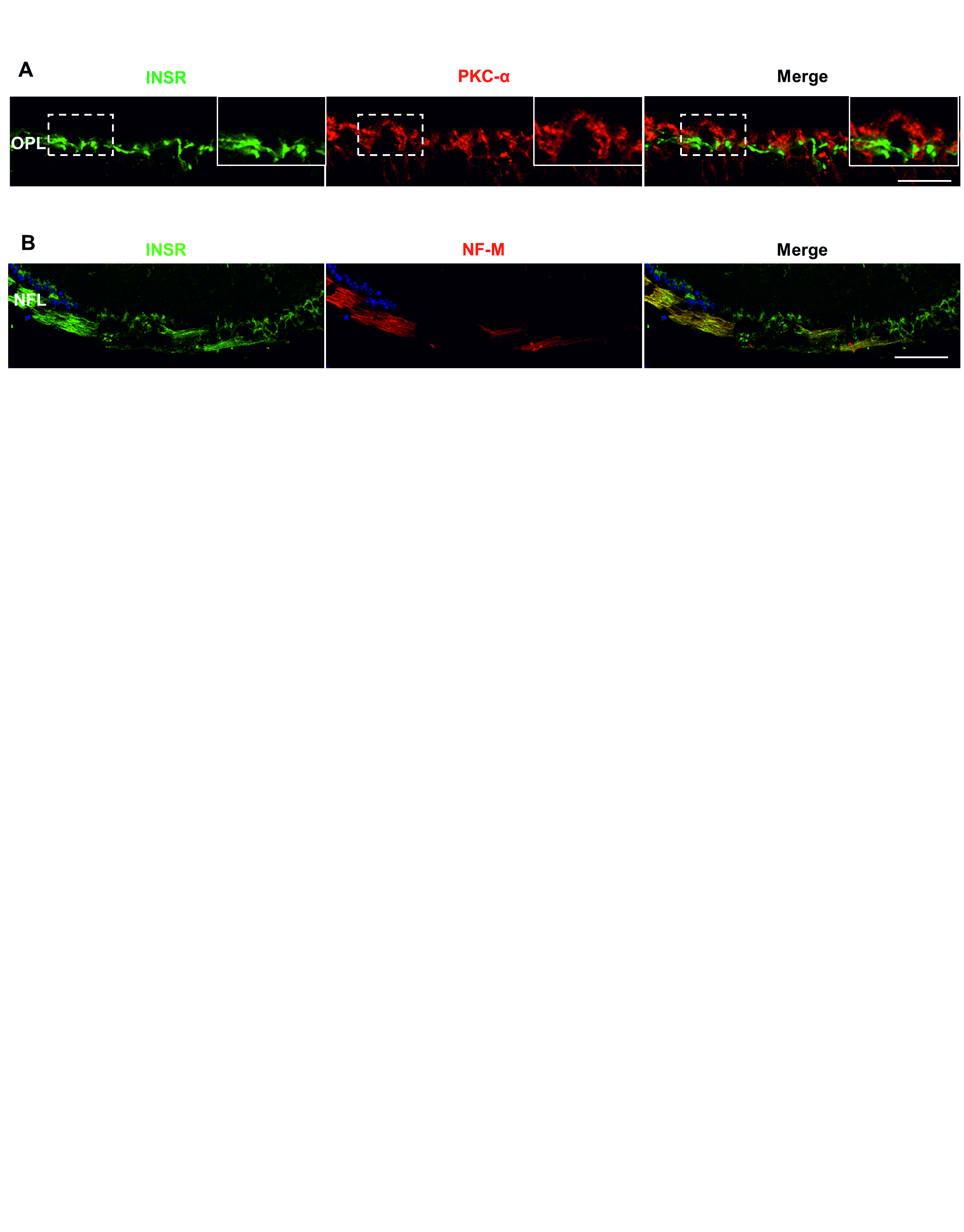

Supplement: Supplementary file 4 — Supplementary Figure S2 [file 41419_2022_4839_MOESM4_ESM.tif]

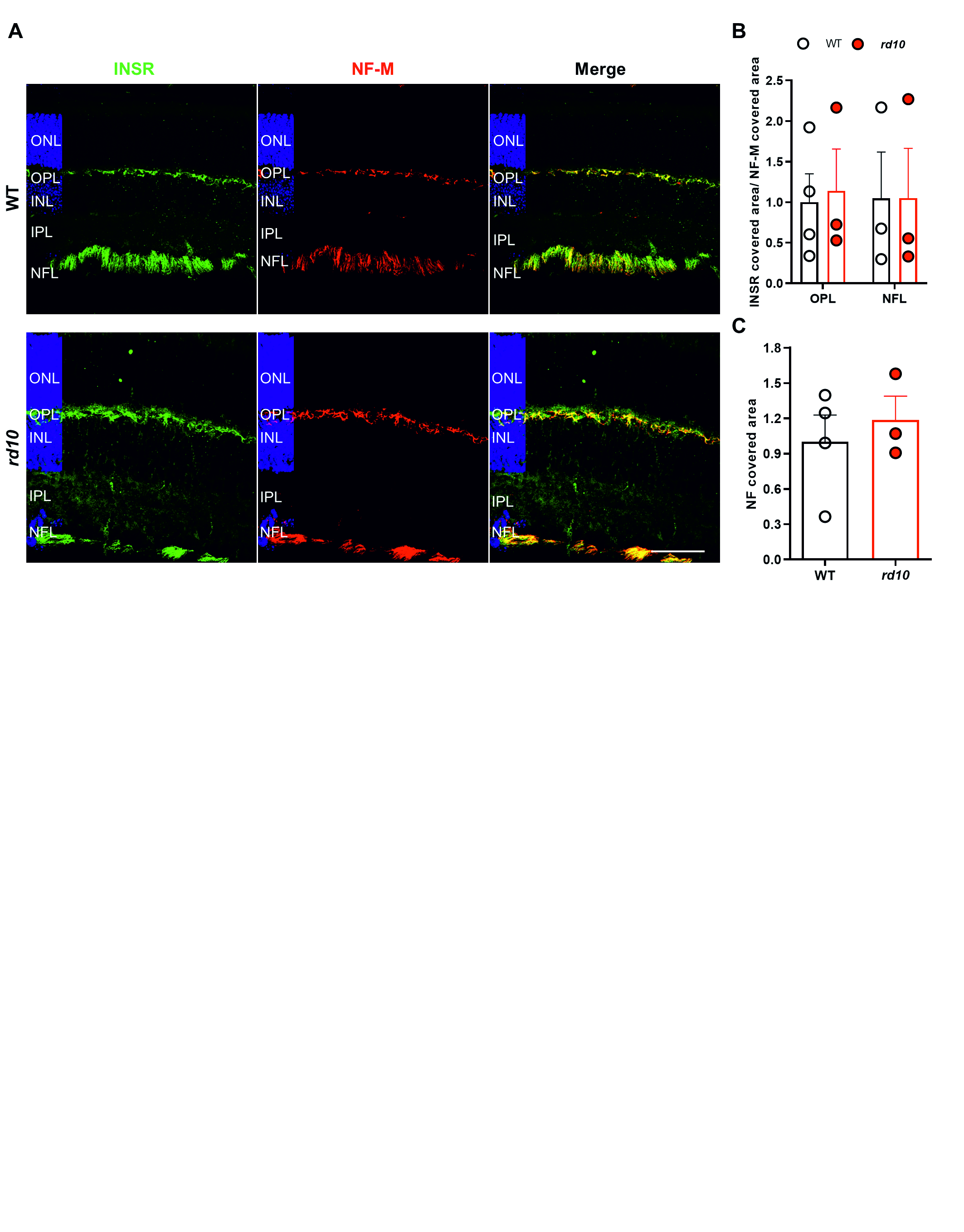

Supplement: Supplementary file 5 — Supplementary Figure S3 [file 41419_2022_4839_MOESM5_ESM.tif]

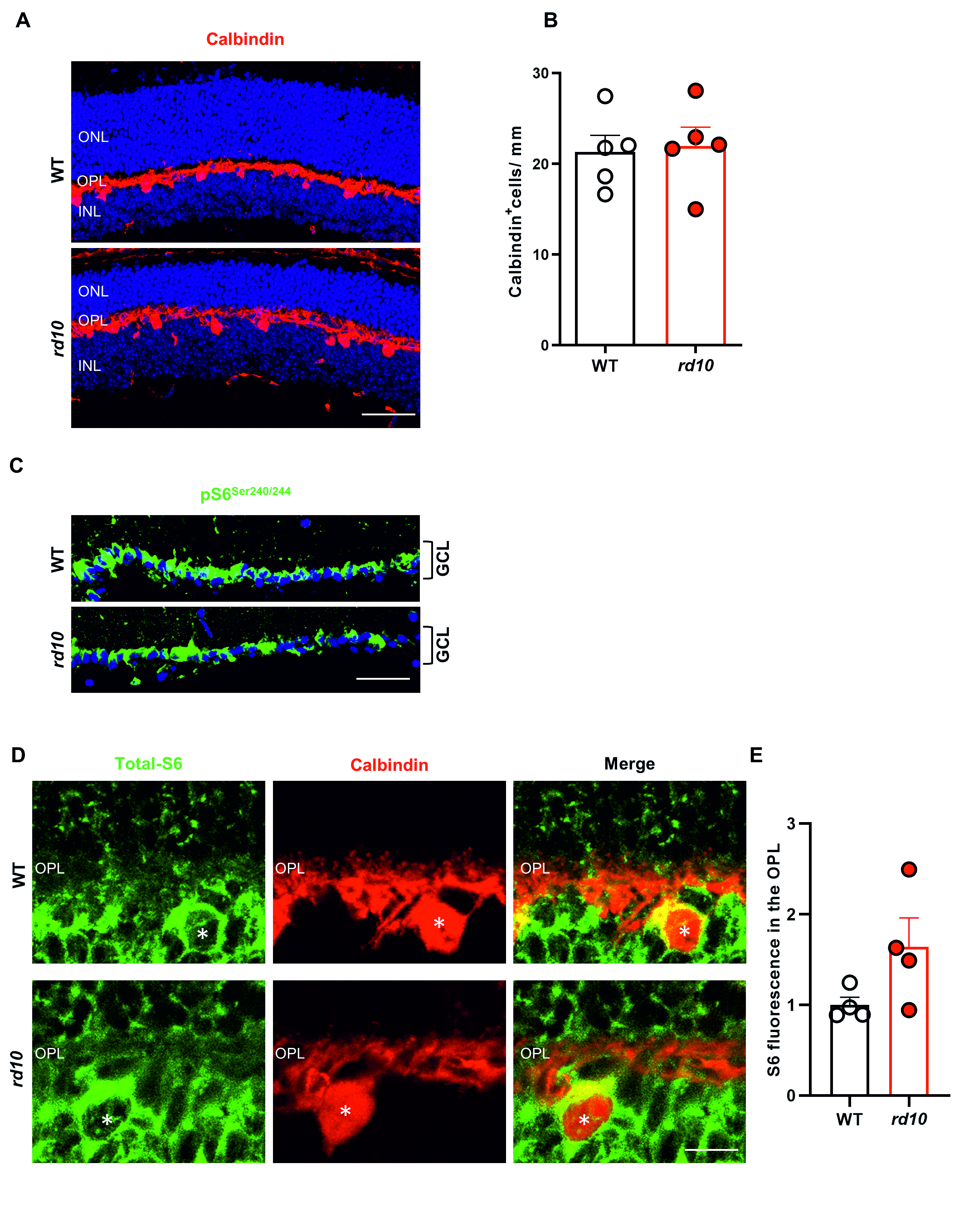

Supplement: Supplementary file 6 — Supplementary Figure S4 [file 41419_2022_4839_MOESM6_ESM.tif]

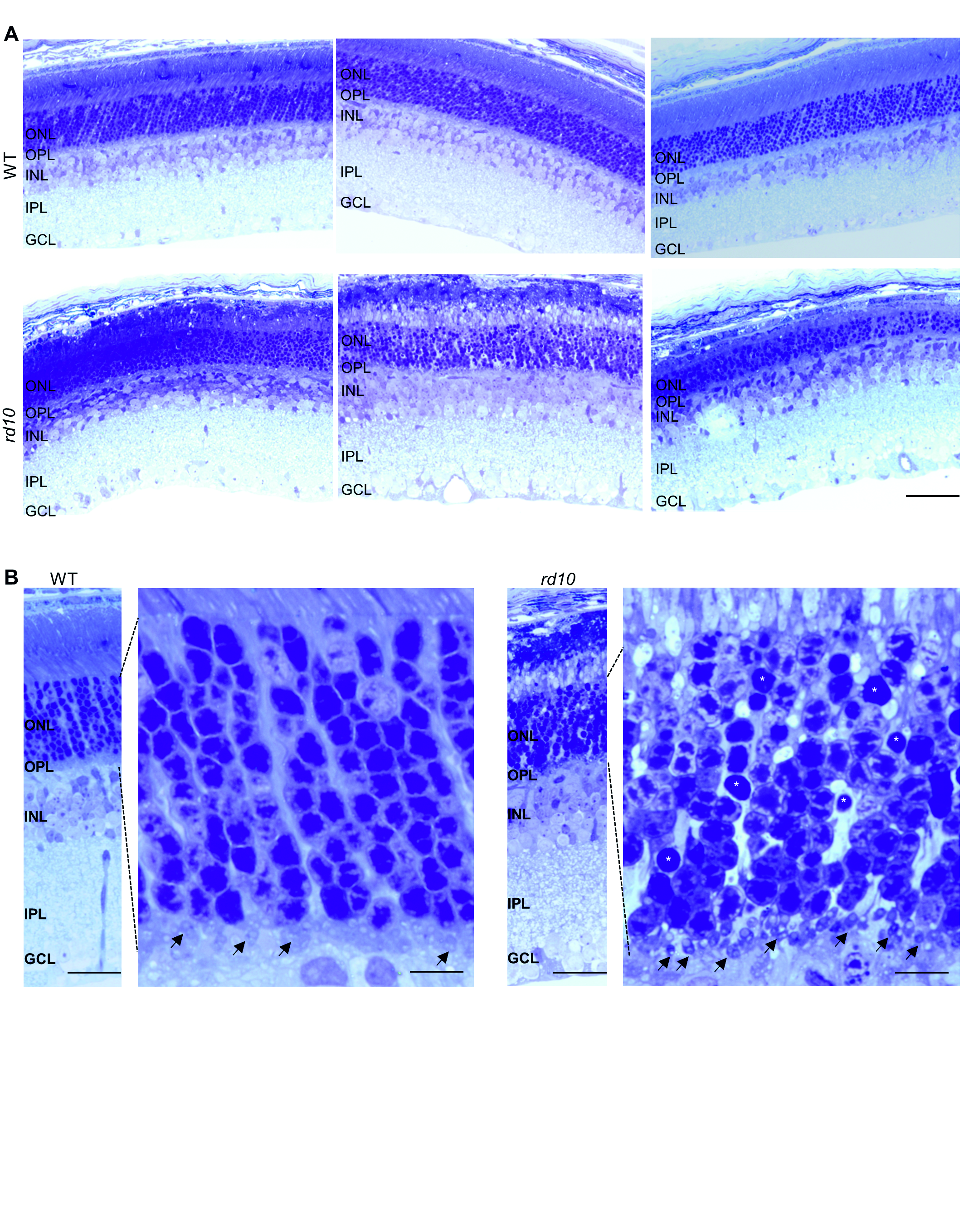

Supplement: Supplementary file 7 — Supplementary Figure S5 [file 41419_2022_4839_MOESM7_ESM.tif]

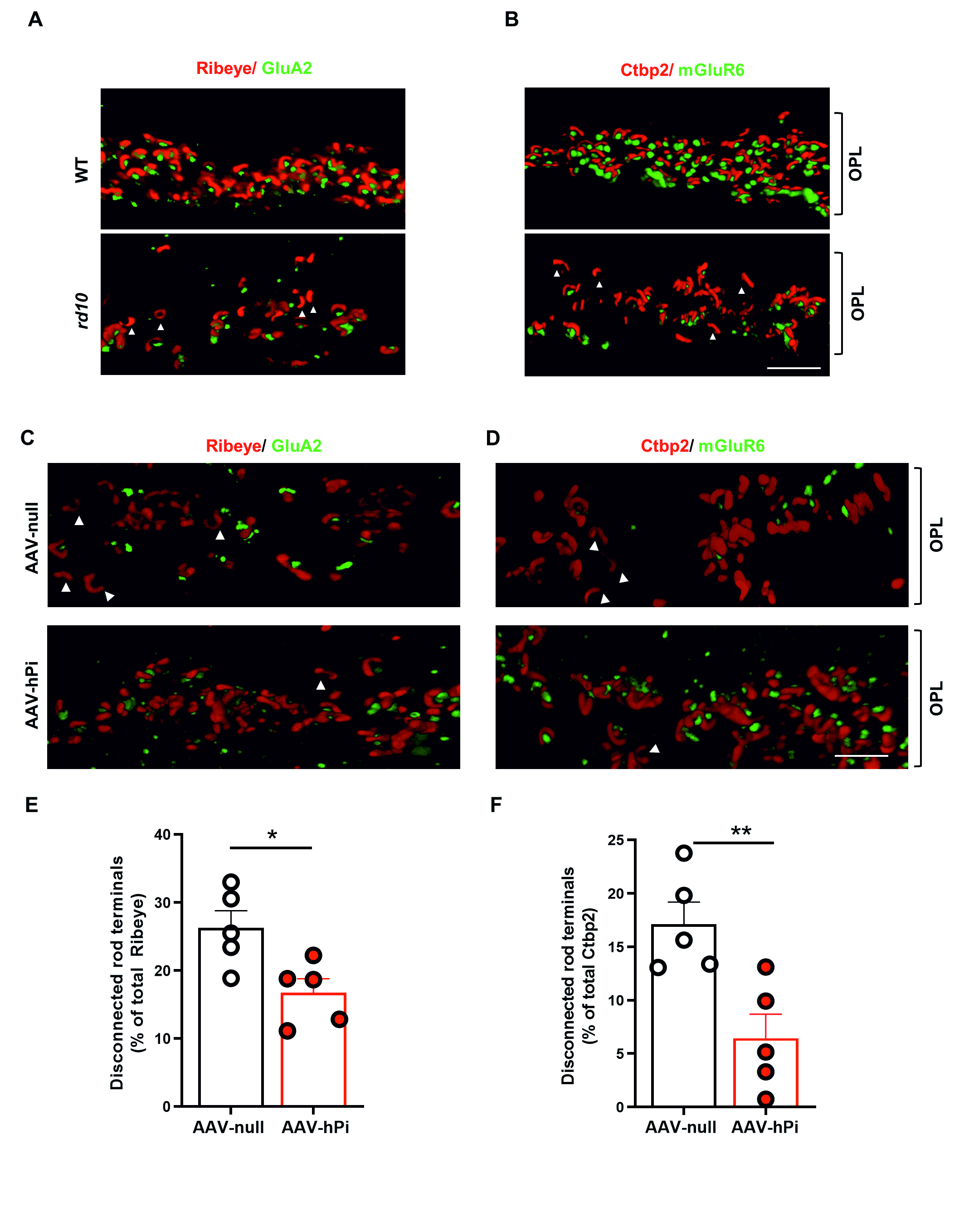

Supplement: Supplementary file 8 — Supplementary Figure S6 [file 41419_2022_4839_MOESM8_ESM.tif]

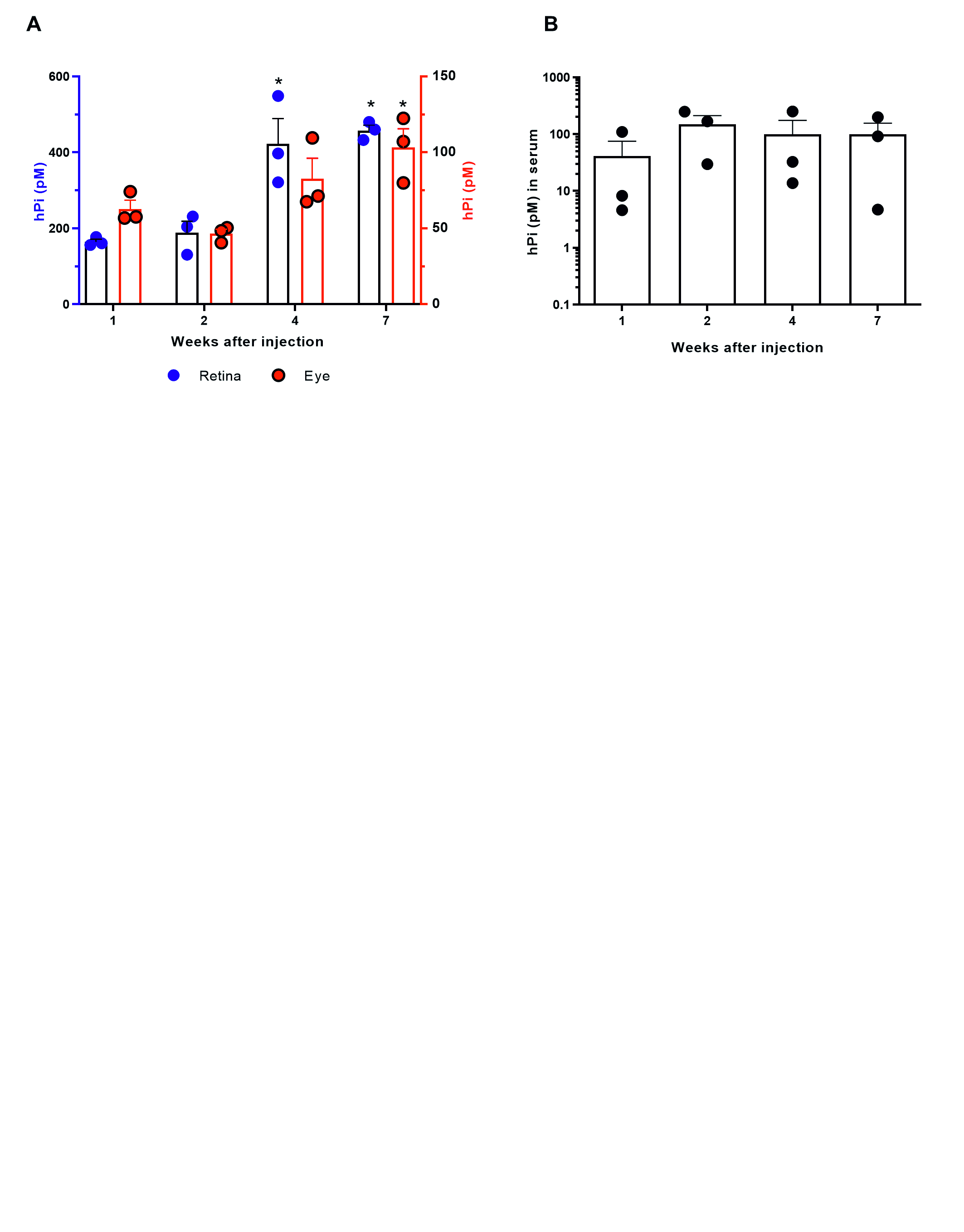

Supplement: Supplementary file 9 — Supplementary Figure S7 [file 41419_2022_4839_MOESM9_ESM.tif]

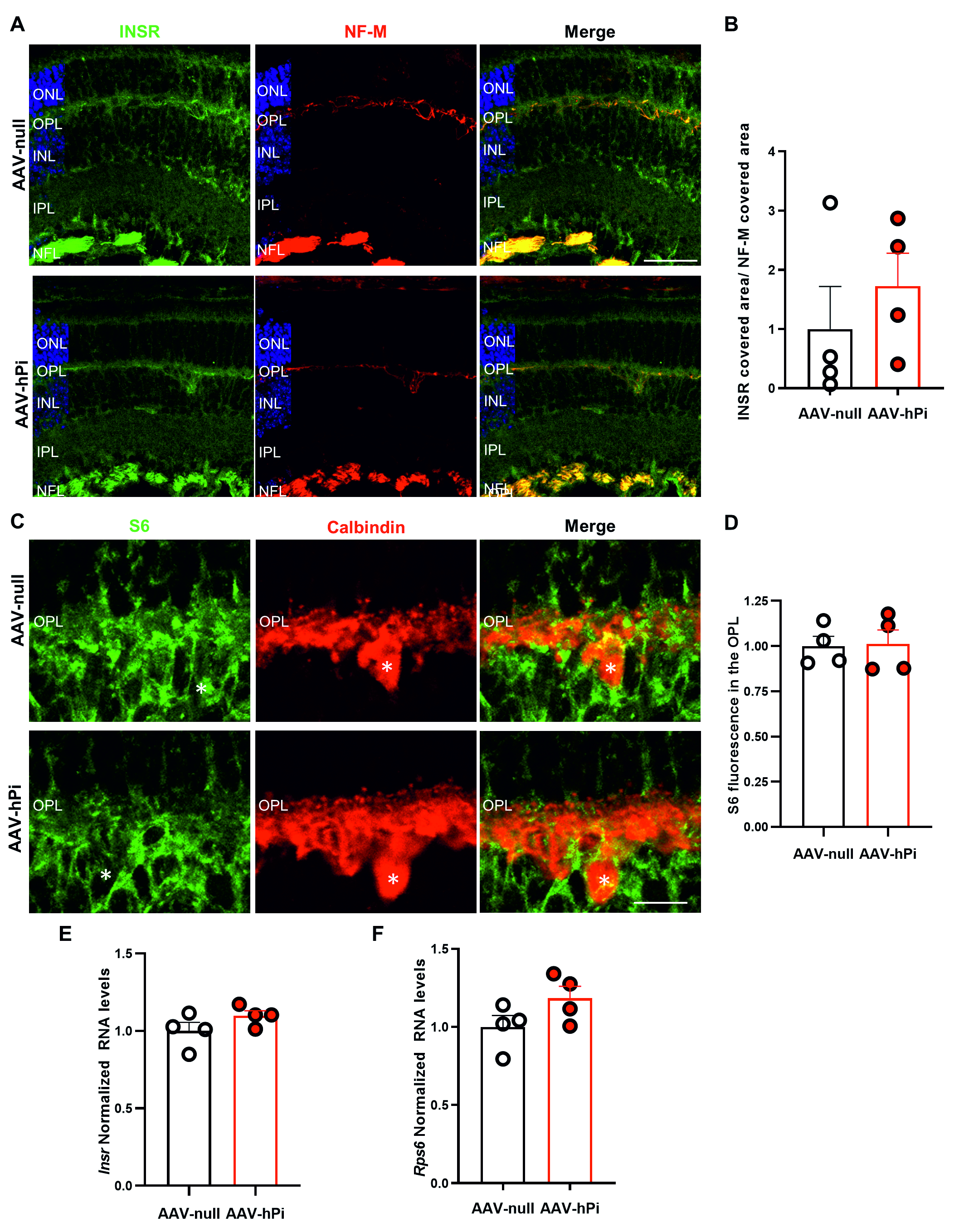

Supplement: Supplementary file 10 — Supplementary Figure S8 [file 41419_2022_4839_MOESM10_ESM.tif]

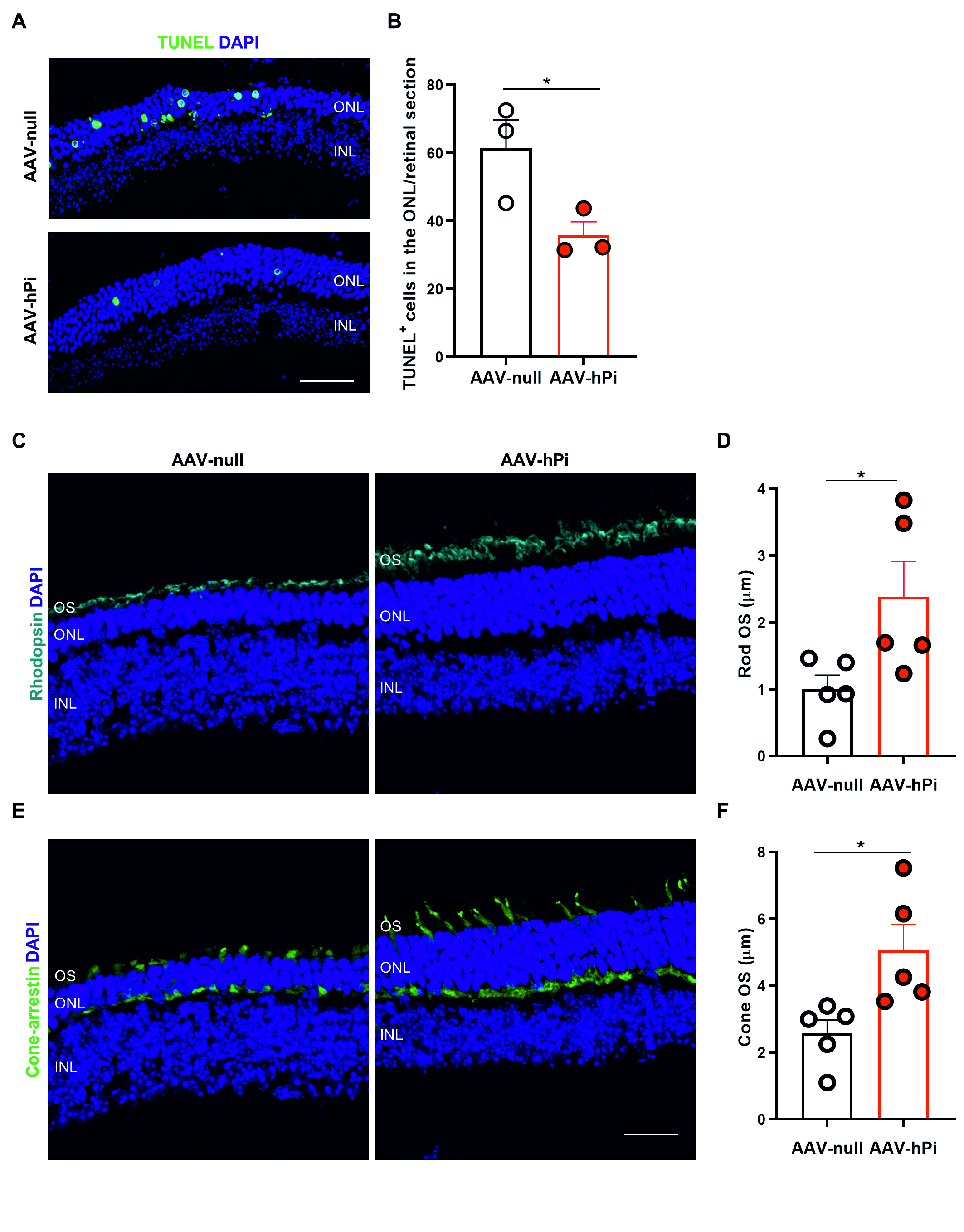

Supplement: Supplementary file 11 — Supplementary Figure S9 [file 41419_2022_4839_MOESM11_ESM.tif]

## Slide 1
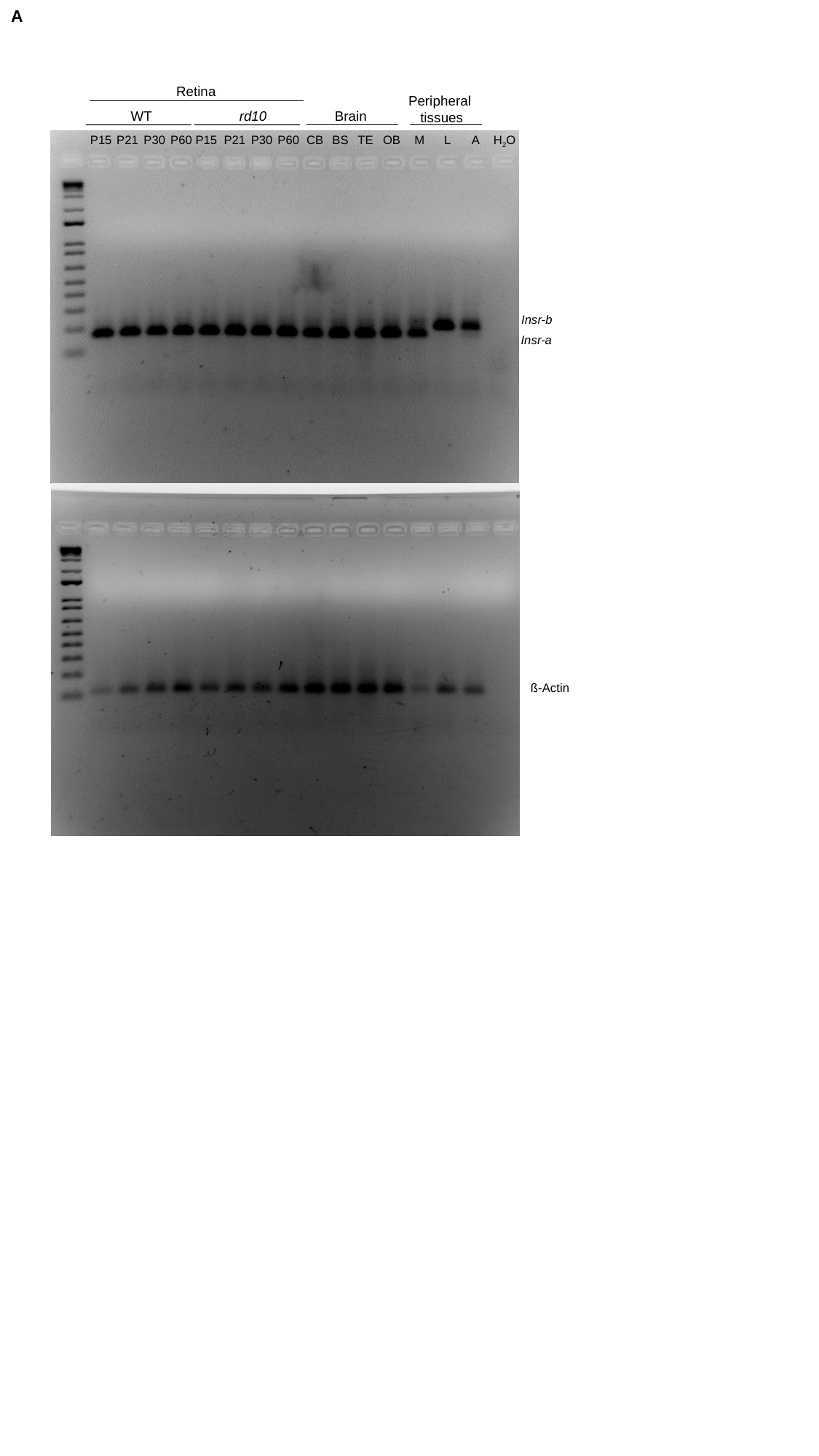

A
Retina
Peripheral
tissues
WT
rd10
Brain
P15
P21
P30
P60
P15
P21
P30
P60
CB
BS
TE
OB
M
L
A
H2O
Insr-b
Insr-a
ß-Actin

Supplement: Supplementary file 12 — Original Data - 1A [file 41419_2022_4839_MOESM12_ESM.pptx]
